# Spin-orbit coupling induced splitting of Yu-Shiba-Rusinov states in antiferromagnetic dimers

**DOI:** 10.1038/s41467-021-22261-6

**Published:** 2021-04-01

**Authors:** Philip Beck, Lucas Schneider, Levente Rózsa, Krisztián Palotás, András Lászlóffy, László Szunyogh, Jens Wiebe, Roland Wiesendanger

**Affiliations:** 1 Department of Physics, University of Hamburg, Hamburg, Germany; 2 Department of Physics, University of Konstanz, Konstanz, Germany; 3 Institute for Solid State Physics and Optics, Wigner Research Center for Physics, Budapest, Hungary; 4 MTA-SZTE Reaction Kinetics and Surface Chemistry Research Group, University of Szeged, Szeged, Hungary; 5 Department of Theoretical Physics, Budapest University of Technology and Economics, Budapest, Hungary; 6 MTA-BME Condensed Matter Research Group, Budapest University of Technology and Economics, Budapest, Hungary

**Keywords:** Magnetic properties and materials, Superconducting properties and materials, Topological defects

## Abstract

Magnetic atoms coupled to the Cooper pairs of a superconductor induce Yu-Shiba-Rusinov states (in short Shiba states). In the presence of sufficiently strong spin-orbit coupling, the bands formed by hybridization of the Shiba states in ensembles of such atoms can support low-dimensional topological superconductivity with Majorana bound states localized on the ensembles’ edges. Yet, the role of spin-orbit coupling for the hybridization of Shiba states in dimers of magnetic atoms, the building blocks for such systems, is largely unexplored. Here, we reveal the evolution of hybridized multi-orbital Shiba states from a single Mn adatom to artificially constructed ferromagnetically and antiferromagnetically coupled Mn dimers placed on a Nb(110) surface. Upon dimer formation, the atomic Shiba orbitals split for both types of magnetic alignment. Our theoretical calculations attribute the unexpected splitting in antiferromagnetic dimers to spin-orbit coupling and broken inversion symmetry at the surface. Our observations point out the relevance of previously unconsidered factors on the formation of Shiba bands and their topological classification.

## Abstract (web summary)

The influence of spin-orbit coupling on the hybridization of Shiba states in dimers of magnetic atoms on superconducting surfaces remains unexplored. Here, the authors reveal a splitting of atomic Shiba orbitals due to spin-orbit coupling and broken inversion symmetry in antiferromagnetically coupled Mn dimers placed on a Nb(110) surface.

## Introduction

The interplay between spin–orbit coupling (SOC), magnetism, and superconductivity has been extensively studied in recent years due to their applications in quantum computation, particularly concerning the realization of topological qubits based on Majorana bound states (MBS). Evidence of MBS that can exist on the edges of topological superconductors have been reported in various systems involving strong SOC, ranging from semiconductor nanowires proximity coupled to *s*-wave superconductors^1–4^, over magnetic vortex cores in topological superconductors^5,6^, to one-^7–11^ and two-dimensional^12–14^ magnetic nanostructures on s-wave superconductors. Promising building blocks for the latter systems are states formed by the hybridization of Yu–Shiba–Rusinov excitations (referred to as Shiba states)^15–17^ which lead to the emergence of so-called Shiba bands in nanostructures. Shiba states are induced in the vicinity of magnetic impurities embedded in or adsorbed on the surface of a superconductor via a potential that locally breaks Cooper pairs. Aiming at tailoring the Shiba bands for topological superconductivity, experimental work has focused on investigations of the Shiba states of single magnetic impurities on superconducting substrates^18–26^ and of coupled dimers of such impurities^27–30^.

In dimers with spacings less than the lateral extent of the Shiba states, the bound states are expected to hybridize. As calculated in ref. ^17^, there is a fundamental difference between ferromagnetically (FM) and antiferromagnetically (AFM) aligned dimers. In FM dimers, the states strongly hybridize and split into a symmetric and an antisymmetric linear combination of the single-impurity Shiba states. In contrast, for AFM alignment, the hybridization is expected to be weaker since quasiparticles of opposite spin are scattered preferentially by the two impurities, which leads to a smaller shift in the Shiba state energies. Importantly, the two Shiba states remain degenerate in a perfectly AFM-aligned dimer, since exchanging the positions of the two impurities while simultaneously switching the spin directions is a symmetry of the system^31–33^. Experimental results have partially confirmed this picture by observing the presence and the absence of the splitting in dimers which have been identified as FM-aligned and AFM-aligned in density-functional theory calculations, respectively^28^. Accordingly, in the absence of information about the exchange interaction between the localized spins^27,30^, it was argued that the observation of the splitting of Shiba states is sufficient to exclude an AFM coupling. All of these experimental observations have been explained based on the theoretical framework formulated by Yu, Shiba, and Rusinov^15–17^. This theory does not take into account SOC, and its influence on the Shiba states has been considered in surprisingly few works so far^34,35^. Over the recent decades, a plethora of novel phenomena in solid-state physics has been demonstrated to arise due to the combination of SOC with inversion-symmetry breaking. These include the emergence of Rashba-split surface states in the electronic structure^36^; the mechanism of the Dzyaloshinsky–Moriya interaction^37,38^, which gives rise to chiral non-collinear magnetic configurations^39–41^; the formation of MBS in magnetic chains proximity coupled to a superconductor^42^; and the presence of the crystal anomalous Hall effect in collinear antiferromagnets^43^.

Here, we reveal a so far unconsidered mechanism of Shiba state hybridization caused by SOC in noncentrosymmetric systems. We present a scanning tunneling spectroscopy (STS) study of the multi-orbital Shiba states of single Mn adatoms and Mn dimers on Nb(110). Using the tip of a scanning tunneling microscope (STM) to artificially construct dimers, we vary interatomic orientations and spacings. We identify dimers both with FM and AFM alignments based on spin-polarized measurements. Regardless of the relative orientation of the spins, we observe shifted and split Shiba states in the Mn dimers. However, for the AFM case, the spatial distributions of their wavefunctions no longer clearly resemble the usual symmetric and antisymmetric combinations of the single-impurity Shiba states that are found for the FM case. Our theoretical calculations demonstrate that taking into account SOC and inversion-symmetry breaking is necessary for lifting the twofold degeneracy of Shiba states in AFM-oriented dimers. We argue that considering this phenomenon is essential for understanding the subgap excitations in artificially designed magnetic nanostructures at the surfaces of superconductors.

## Results

### Multi-orbital Shiba states of single Mn adatoms

**Fig. 1:**
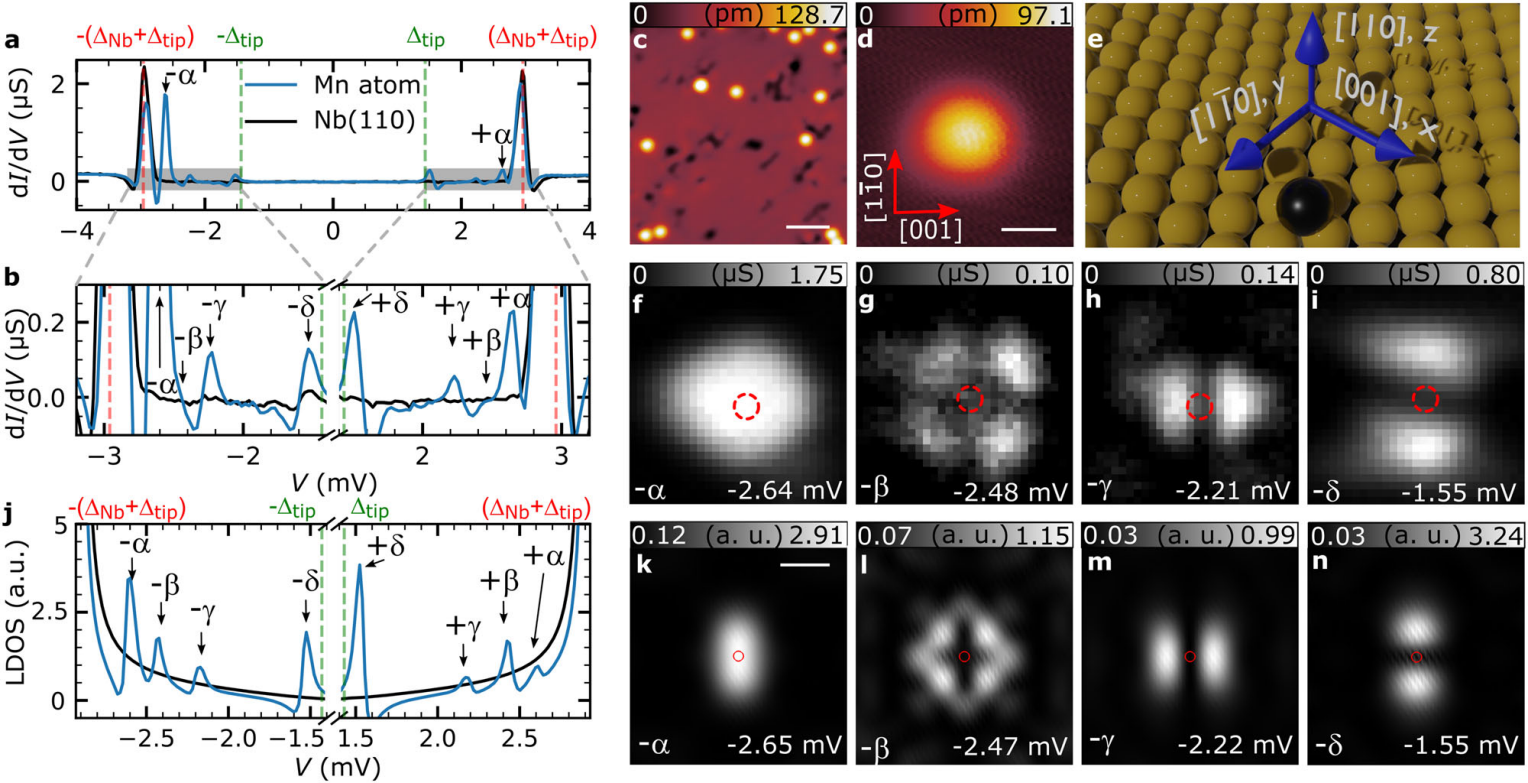
Shiba states of single Mn adatoms on Nb(110). **a** d*I*/d*V* spectra obtained on bare Nb(110) (black) and over a Mn adatom (blue). Red and green vertical lines mark the positions of the coherence peaks ±(Δ_Nb_ + Δ_tip_) and the tip gap Δ_tip_, respectively. **b** Magnification of the spectra shown in panel **a**. The coherence peaks and the two peaks with the largest intensity are left out for the sake of visibility. Shiba states are labeled and marked by arrows. **c** Overview STM image of the Mn/Nb(110) sample, where bright protrusions are single Mn adatoms and black depressions correspond to residual oxygen. The white scale bar has a length of 3 nm. (*V*_bias_ = 100 mV and *I* = 200 pA). **d** STM image of a single Mn adatom, which was used for recording the spectra in **a** and **b**, as well as for the d*I*/d*V* maps in **f**–**i**. The white scale bar has a length of 500 pm and the red arrows point along two high symmetry directions $\left[ {1\bar 10} \right]$ and [001] of the Nb(110) surface. The directions also apply to panels **f**–**i** and **k**–**n**. **e** 3D rendered illustration of the position of the single Mn adatom (black sphere) with respect to the Nb substrate atoms (yellow spheres), including the high-symmetry directions indicated by blue arrows with their nomenclature. **f**–**i** d*I*/d*V* maps measured at the given bias voltages of the Shiba states marked in **a** and **b** and in the area of **d** in the vicinity of the single Mn adatom. The d*I*/d*V* maps taken at the Shiba peaks on the positive bias side are shown in Supplementary Fig. 5. **j** Calculated LDOS inside the superconducting gap convoluted with the DOS of the superconducting tip ($\pm \Delta _{{\mathrm{{tip}}}} = 1.43$ mV) at the position of the Mn adatom (blue) and on the bare superconductor (black). **k**–**n** Calculated two-dimensional maps of the LDOS at the bias voltages indicated in the figures. The white scale bar has a length of 500 pm. Red circles in **f**–**i** and **k**–**n** highlight the position of the adatom.

Mn atoms were deposited on a clean Nb(110) surface (Fig. 1c, see the “Methods” section) and are adsorbed on the hollow site in the center of four Nb atoms (Fig. 1e). First, we revisit the d*I*/d*V* spectra of the single adatoms with a considerably better energy resolution than reported previously^18^ (Fig. 1a, b) which is achieved using superconducting tips and lower temperatures (see the “Methods” section). Compared to the spectra taken on bare Nb(110), they reveal four pairs of additional resonances inside the superconductor’s energy gap, one at positive and one at negative bias symmetrically with respect to the Fermi energy *E*_F_ (*V* = 0 V), which we label ±α, ±β, ±γ, and ±δ. The resonance labeled ±β is only visible as a shoulder of the ±α peak. Using d*I*/d*V* maps, we determine the spatial distribution^19,20,30^ of these four resonances, revealing an astonishing resemblance to the shape of the well-known atomic *d*-orbitals (Fig. 1f–i, see the corresponding maps of the positive bias partner in Supplementary Fig. 5). The energetically highest and most intense state ±α has a circular shape and faint lobes along the [001] (*x*) and $[1\bar 10]$ (*y*) direction (Fig. 1f and Supplementary Fig. 5a), which hints towards an origin in the $d_{z^2}$ or the $d_{x^2 - y^2}$ orbital. The three other states have *d*_*xy*_-like (±β), *d*_*xz*_-like (±γ) and *d*_*yz*_-like (±δ) shapes (Fig. 1g–i and Supplementary Fig. 5b–d). Moving away from the adatom center, the spectral intensities of the states decrease rapidly to a tenth of their maximum values within a range of 1 nm, and only very weak oscillations of the spectral intensity can be observed at larger distances (Supplementary Fig. 5e–l). We correspondingly assign the states to multi-orbital Shiba states^19,20,30^ formed by the Mn adatom as can be understood based on the following theoretical model. The free-standing Mn atom has a spin of $S = \frac{5}{2}$ in the ground state according to Hund’s first rule, with each of its five degenerate *d-*orbitals being singly occupied. As the atom is placed on the Nb(110) surface, its atomic states hybridize with the substrate, and the degeneracy of the states is lifted by the crystal field. Based on symmetry arguments (see Supplementary Note 10 for details), it can be concluded that the *d*_*xy*_, *d*_*xz*_ and *d*_*yz*_ orbitals function as three separate scattering channels of different shapes and strengths acting on the quasiparticles of the superconductors. The $d_{z^2}$ and $d_{x^2 - y^2}$ orbitals hybridize and form two scattering channels, only one of which leads to an observable Shiba state in the experiments, while the other may be hidden in the coherence peaks. The shapes of these scattering channels were extracted from ab initio calculations performed for the Mn adatom on Nb(110), based on the procedure described in ref. ^20^ (see the “Methods” section and Supplementary Note 6). The strengths of the non-magnetic (*K*) and magnetic (*JS*/2) scattering were determined in such a way that the calculated local density of states (LDOS) at the position of the adatom, presented in Fig. 1j, resembles the experimental d*I*/d*V* spectrum, including the energy positions of the peaks and the particle–hole asymmetry in intensity between Shiba pairs located at positive and negative bias, respectively. The values of *K* and *JS*/2 are listed in Supplementary Table 1. The spatial distributions of the LDOS at the Shiba resonance energies, illustrated in Fig. 1k–n (see Supplementary Fig. 16 for the positive-energy states), reasonably agree with the experimental data (Fig. 1f–i) demonstrating the robustness of the theoretical model.

### Hybridization of Shiba states in ferromagnetic dimers

**Fig. 2:**
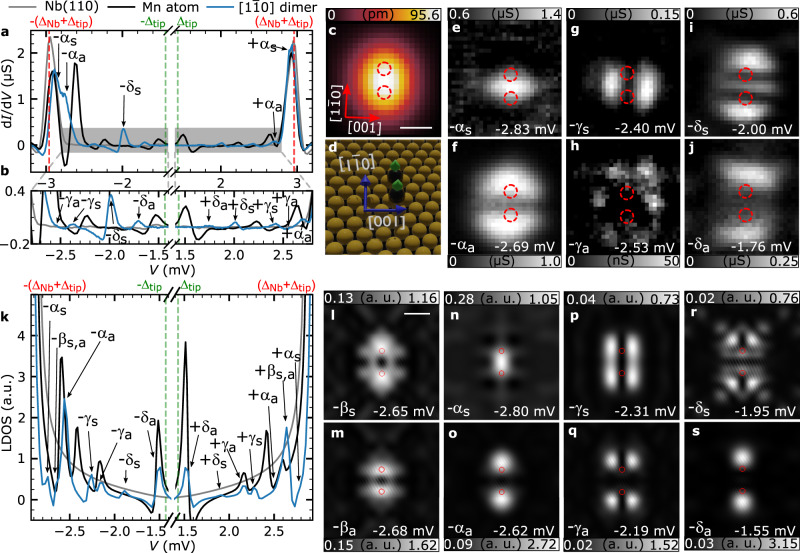
Hybridized Shiba states in a FM-coupled $\sqrt 2 {a} - [1\bar 10]$ Mn dimer. **a** d*I*/d*V* spectrum taken between the two atoms of a $\sqrt 2 {a} - [1\bar 10]$ dimer (blue) with reference spectra for the substrate (gray) and a single Mn adatom (black). **b** Magnification of the spectra shown in panel **a**. Shiba states are labeled and marked by arrows. Vertical lines mark sample and tip gaps. **c** Small-scale STM image of a $\sqrt 2 {a} - [1\bar 10]$ Mn dimer. The white scale bar has a length of 500 pm (*V*_bias_ =6 mV and *I*=1 nA). The directions and the scale bar also apply to panels **e**–**j**. **d** 3D rendered illustration of the positions of the two Mn adatoms (black spheres) in the investigated dimer with respect to the Nb substrate atoms (yellow spheres), including the spin structure indicated by green arrows. **e**–**j** d*I*/d*V* maps measured on the dimer in the same area shown in panel **c** for the given bias voltages where a peak/shoulder is visible in the spectrum in **a** and **b**. The d*I*/d*V* maps taken at the Shiba peaks on the positive bias side are shown in Supplementary Fig. 6. LDOS calculated between the two atoms of the FM-aligned $\sqrt 2 {a} - [1\bar 10]$ dimer (blue), on a single adatom (black) and on the bare superconductor (gray) convoluted with the DOS of the superconducting tip. **l**–**s** Calculated two-dimensional maps of the LDOS at the indicated bias voltages. The white scale bar has a length of 500 pm; crystallographic directions as in panel **c**. Red circles in **c**, **e**–**j** and **l**–**s** denote the locations of the Mn adatoms in the dimer.

We now turn to the investigation of the Shiba states in Mn dimers. We can tune the magnetic exchange interaction between FM and AFM by laterally manipulating one of the adatoms with the STM tip (see the “Methods” section), thereby varying the crystallographic direction and interatomic spacing. The positions of the two atoms in all manipulated dimers have been determined as described in the “Methods” section, Supplementary Note 2 and Supplementary Fig. 2. We first consider the close-packed dimer along the $\left[ {1\bar 10} \right]$ direction (see Fig. 2c, d) denoted as $\sqrt 2 {a} - \left[ {1\bar 10} \right]$ dimer, where *a* = 329.4 pm is the nearest-neighbor spacing along [001], corresponding to the bulk lattice constant of bcc Nb. Spin-polarized STM (SP-STM) measurements on close-packed chains in this direction (Supplementary Note 4 and Supplementary Fig. 4) indicate that the spins in this dimer are FM-aligned (Fig. 2d). The d*I*/d*V* spectrum taken on top of the dimer (Fig. 2a and b, blue lines) shows six pairs of Shiba states in contrast to the four pairs of states observed for the single adatom (black line). By comparing their energies and spatial distributions in d*I*/d*V* maps (Fig. 2e–j and Supplementary Fig. 6) with the energies and shapes of the single-adatom Shiba states (Fig. 1b, f–i), we conjecture that these six states can be sorted out into three pairs upon hybridization of single-adatom Shiba states, one of the ±α (Fig. 2e, f and Supplementary Fig. 6a, b), one of the ±γ (Fig. 2g, h and Supplementary Fig. 6c, d), and one of the ±δ (Fig. 2i,j and Supplementary Fig. 6e, f) state. Moreover, for each of these three pairs, one state has maxima in the *xz* plane in the center between the two Mn adatoms of the dimer (Fig. 2e, g, i and Supplementary Fig. 6a, d, e) while the other approximately has a nodal line in that plane (Fig. 2f, h, j and Supplementary Fig. 6b, c, f). Therefore, we tentatively assign them to symmetric (s) and antisymmetric (a) linear combinations of the single-adatom ±α, ±γ, and ±δ Shiba states^17,31,32^. Hybridized states of the type ±β could not be identified in the dimer, presumably because of their weak intensity as observed for the single adatom.

Model calculations (see the “Methods” section) using the scattering channel parameters determined for the adatom support these conclusions (Fig. 2k–s and Supplementary Fig. 17). There are eight pairs of Shiba states of the dimer visible as peaks in the calculated LDOS (Fig. 2k), which may be separated into symmetric and antisymmetric combinations with respect to the *xz* mirror plane, as it was performed for the experimental images. Based on the spatial profiles of the states (Fig. 2l–s) we denote them as ±α_s_ and ±α_a_ (Fig. 2n, o), ±γ_s_ and ±γ_a_ (Fig. 2p, q), as well as ±δ_s_ and ±δ_a_ (Fig. 2r, s), respectively. The two additional states (Fig. 2l, m) which are not observed in the experiment are assigned to the ±β_s_ and ±β_a_ states, although their spatial profile also shows similarities with the α states being close by in energy. The latter states were separated from each other by performing calculations with a higher energy resolution than shown in Fig. 2k. Comparing experimental and theoretical results of the energetic shifts of the hybridized Shiba states relative to the single-adatom states and the splitting of symmetric and antisymmetric states (Table 1), we can conclude that the model reproduces the experimental results reasonably well.

**Table 1:** Comparison of energetic shifts and splittings of hybridized Shiba states in a FM-coupled $\sqrt 2 {a} - [1\bar 10]$ Mn dimer.

Shiba state	Experiment		Theory	
	Shift (μV)	Splitting (μV)	Shift (μV)	Splitting (μV)
α	+120	+140	+60	+180
β	−	−	+195	−30
γ	+260	−130	+30	+120
δ	+330	+240	+200	+400

The energetic shifts were calculated from the experimental and theoretical results by $\frac{1}{2}\left( {\left| {E_{n_{\mathrm{{a}}}}} \right| + \left| {E_{n_{\mathrm{{s}}}}} \right|} \right) - |E_n|$, where $E_{n}$ are the energetic positions of the single-adatom Shiba states $n \in {\mathrm{{(\alpha ,\beta ,\gamma ,\delta )}}}$, and $E_{n_{\mathrm{{a}}}}$ and $E_{n_{\mathrm{{s}}}}$ are the energies of the respective antisymmetric and symmetric Shiba states in the dimer. The splittings of hybridized Shiba states are calculated by $\left| {E_{n_{\mathrm{{s}}}}} \right| - \left| {E_{n_{\mathrm{{a}}}}} \right|$.

### Hybridization of Shiba states in antiferromagnetic dimers

**Fig. 3:**
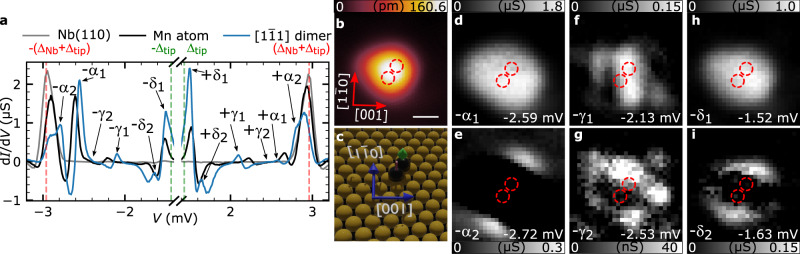
STS of hybridized Shiba states in an AFM-coupled $\sqrt 3 {a}/2 - [1\bar 11]$ Mn dimer. **a** d*I*/d*V* spectrum taken between the two atoms of a $\sqrt 3 {a}/2 - [1\bar 11]$ Mn dimer (blue). A reference spectrum taken on a single Mn adatom (black) and a patch of clean Nb(110) (gray) are pasted to the background. Shiba states are labeled and marked by arrows. **b** STM image of a $\sqrt 3 {a}/2 - [1\bar 11]$ Mn dimer. The white scale bar has a length of 500 pm and also applies to panels **d**–**i** (*V*_bias_ = 6 mV and *I* = 1 nA). **c** 3D rendered illustration of the positions of the two Mn adatoms (black spheres) in the investigated dimer with respect to the Nb substrate atoms (yellow spheres), including the spin structure indicated by green and red arrows. **d**–**i** d*I*/d*V* maps measured on the dimer shown in panel **b** for the given bias voltages where a peak/shoulder is visible in the spectrum in **a**. The d*I*/d*V* maps taken at the Shiba peaks on the positive bias side are shown in Supplementary Fig. 7.) The same orientation from panel **b** applies to these maps. Red circles in **b** and **d**–**i** denote the locations of the Mn adatoms in the dimer.

To investigate the effect of the spin configuration on Shiba states of a dimer and to check for the reported absence of split Shiba states in AFM-coupled dimers^31,32^, we study the nearest-neighbor dimer constructed along the diagonal of the centered rectangular unit cell (denoted as $\sqrt 3 {a}/2 - [ {1\bar 11} ]$ dimer, Fig. 3b, c). Recent SP-STM measurements on short chains of close-packed Mn adatoms along this direction^44^ indicate that the exchange interaction between the adatoms in this dimer is AFM (Fig. 3c). This is further supported by our calculations based on the fully relativistic screened Korringa–Kohn–Rostoker (KKR) method^45^, which reveal an AFM ground state for the dimer (Supplementary Table 3). Surprisingly, the d*I*/d*V* spectrum taken on the dimer (Fig. 3a, blue line) displays six pairs of sharp peaks, implying the hybridization and energetic splitting of the single-adatom Shiba states. From the spatial distributions of the d*I*/d*V* maps taken at the peak positions (Fig. 3d–i and Supplementary Fig. 7) and by following similar arguments as for the $\sqrt 2 {a} - [1\bar 10]$ dimer given above, we conclude that also for this $\sqrt 3 {a}/2 - [ {1\bar 11} ]$ dimer the ±α, ±γ, and ±δ Shiba states of the single Mn adatom split into pairs of hybridized states. However, while the pairs ±α_1_ and ±α_2_ (Fig. 3d, e and Supplementary Fig. 7a, b) as well as ±δ_1_ and ±δ_2_ (Fig. 3h, i and Supplementary Fig. 7e, f) still resemble symmetric and antisymmetric linear combinations of the single-adatom Shiba states, the situation is not as obvious for the pair ±γ_1_ and ±γ_2_ (Fig. 3f, g and Supplementary Fig. 7c, d). Note that the symmetry in this dimer is reduced from *C*_2v_ to *C*_2_ containing only a twofold rotation around the *z* axis, which slightly complicates the assignment of the states. Similarly to the $\sqrt 2 {a} - [1\bar 10]$ dimer, spectroscopic signatures of the possible ±β Shiba orbitals in this dimer could not be identified, presumably because of their reduced intensity. Remarkably, the observed splitting of Shiba states is at least of similar strength for the AFM- as for the FM-coupled dimer (cf. Tables 1 and 2). A Shiba-state splitting of similar strength is observed for the $2{a} - \left[ {001} \right]$ dimer as well (Supplementary Fig. 8) which is also AFM-coupled (Supplementary Notes 4 and 5). For the $2{a} - \left[ {001} \right]$ dimer the split Shiba states likewise no longer simply resemble symmetric and antisymmetric linear combinations of the single-adatom Shiba states (Supplementary Note 5). In contrast, each Shiba state at positive energy appears rather antisymmetric compared to their negative-energy partners which are rather symmetric. Overall, it is surprising to find Shiba state splitting in AFM dimers since theoretical calculations in the seminal paper of Rusinov^17^, extended upon in later works^31,32,46^, predicted that for this type of coupling the Shiba states may shift in energy, but they always remain degenerate.

### Role of SOC for the hybridization of Shiba states

**Fig. 4:**
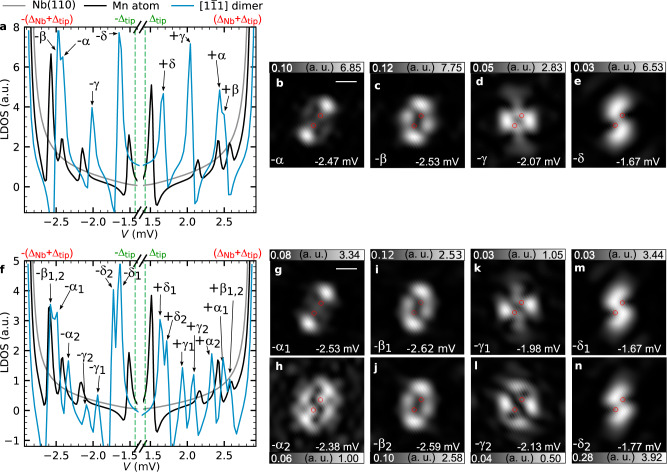
Calculation of hybridized Shiba states in an AFM-coupled $\sqrt 3 {a}/2 - [1\bar 11]$ Mn dimer. LDOS inside the superconducting gap calculated between the two atoms of the out-of-plane AFM-aligned dimer (blue), for a single adatom (black) and on the bare superconductor (gray), **a** without and **f** with taking SOC into account. The spectrum is convoluted with the superconducting DOS of the tip. Shiba states are labeled and marked by arrows. Two-dimensional maps of the LDOS at the indicated bias voltages show the spatial profiles of the states without (**b**–**e**) and with (**g**–**n**) SOC. Red circles denote the positions of the adatoms in the dimer. The white scale bar has a length of 500 pm. The crystallographic axes are the same as in Fig. 3b. Magnetic and non-magnetic scattering parameters with and without SOC are given in Supplementary Tables 1 and 2, respectively.

In order to find a theoretical explanation for this experimental observation, we first discuss the origin of the degeneracy of the Shiba states in AFM-coupled dimers based on symmetry arguments. In the absence of magnetic impurities, the system may be characterized by a Hamiltonian which is invariant under time reversal, represented for spin-1/2 systems by the antiunitary operator $T = {\mathrm{i}}\sigma _4^y\tau ^zK$, where *K* denotes complex conjugation and $\sigma _4^y\tau ^z$ is a spin matrix in Nambu space (see the “Methods” section). According to Kramers’ theorem, *T*^2^ = −1 implies that all eigenstates of this Hamiltonian are pairwise degenerate. The inclusion of the AFM-aligned impurities breaks time-reversal symmetry, but the Hamiltonian remains invariant under the operation *T*_r_ = *TC*_2_, a combination of time reversal and a rotation around the out-of-plane direction by 180° in real space. *T*_r_ effectively also acts as time reversal, since it is an antiunitary symmetry with $T_{\mathrm{r}}^2 = - 1$. This means that Kramers’ theorem still applies in this case, implying a pairwise degeneracy of the Shiba states in the superconductor in particular. This description holds for any AFM-aligned Mn dimer located in the hollow positions on the Nb(110) surface, since they all have a *C*_2_ rotational symmetry if the magnetic configuration is disregarded. The situation is different if SOC is taken into account. Time reversal (*T*) remains a symmetry of the system in the absence of magnetic impurities, but *T*_r_ must be extended since the bulk Hamiltonian is no longer invariant under spin rotation. The spin and orbital degrees of freedom have to be rotated simultaneously, which results in $T_{r\prime } = {\mathrm{TC}}_2{\mathrm{i}}\sigma _4^z\tau ^z$ as an antiunitary symmetry operation for an AFM dimer with out-of-plane spins, which is the case for the $\sqrt 3 {a}/2 - [1\bar 11]$ dimer. For this symmetry one obtains $T_{r\prime }^2 = 1$, meaning that Kramers’ theorem does not apply, and a lifting of the degeneracies is expected. Results of a numerical calculation using material-specific parameters for the AFM $\sqrt 3 {a}/2 - [1\bar 11]$ dimer are displayed in Fig. 4. In the absence of SOC, the LDOS shows the same number of peaks as in the case of the adatom (Fig. 4a), although their energies are shifted. The spatial distributions of these dimer Shiba states are visualized in Fig. 4b–e (see Supplementary Fig. 18a–d for the positive-energy states), where they were assigned the same ±α, ±β, ±γ and ±δ labels based on their relative similarity in energy position and spatial distribution to the Shiba states of the adatom. As expected from the considerations above, if SOC is taken into account (see the “Methods” section), the resonance peaks clearly split in the AFM dimer (Fig. 4f). Since the spatial distributions of the Shiba states in Fig. 4g–n (see Supplementary Fig. 18e–l for the positive-energy states) still show remarkable similarity to the states obtained without SOC (cf. Fig. 4b with g, c with i or j, d with k, and e with m or n) we can assign them to the original states and accordingly name them ±α_1_, ±α_2_, etc. The calculated spatial distributions and splittings including SOC qualitatively agree with the experimental data (cf. Figs. 3d–i and 4g–n and Table 2). In particular, as found in the experiment (Fig. 3f, g and Supplementary Fig. 8c–l) the spatial distributions no longer resemble symmetric and antisymmetric linear combinations of the original single-adatom states. Finally, it is worth mentioning that SOC in the presence of inversion-symmetry breaking also gives rise to the Dzyaloshinsky–Moriya interaction, due to which the spin orientation of the FM-coupled and AFM-coupled dimers might become non-collinear. Based on first-principles calculations using the KKR method, it was found for the $\sqrt 3 {a}/2 - [1\bar 11]$ dimer that this canting from the collinear AFM state is only around 0.5° (see Supplementary Table 3). This degree of non-collinearity would not be sufficiently large to explain the experimentally observed magnitude of the splittings of the peaks without considering SOC directly in the calculation of the Shiba states (Supplementary Note 9).

**Table 2:** Comparison of energetic shifts and splittings of hybridized Shiba states in an AFM-coupled $\sqrt 3 {a}/2 - [1\bar 11]$ Mn dimer.

Shiba state	Experiment		Theory	
	Shift (μV)	Splitting (μV)	Shift (μV)	Splitting (μV)
α	+15	−130	−195	+150
β	−	−	+135	+30
γ	+120	−400	−165	−150
δ	+25	−110	+170	−100

The energetic shifts were calculated from the experimental and theoretical results by $\frac{1}{2}\left( {\left| {{\mathrm{E}}_{{n}_2}} \right| + \left| {{\mathrm{E}}_{{n}_1}} \right|} \right) - \left|{\mathrm{E}}_{n} \right|$, where ${\mathrm{E}}_{n}$ are the energetic positions of the single-adatom Shiba states $n \in ({\upalpha},{\upbeta},{\upgamma},{\updelta})$, and ${\mathrm{E}}_{{n}_2}$ and ${\mathrm{E}}_{{n}_1}$ are the energies of the respective hybridized Shiba states in the dimer. The splittings of hybridized Shiba states are calculated by $\left| {{\mathrm{E}}_{{n}_1}} \right| - \left| {{\mathrm{E}}_{{n}_2}} \right|$.

## Discussion

In conclusion, we demonstrated that the Shiba states of a Mn adatom on the Nb(110) substrate hybridize and split in dimers with considerable overlap between the states. This can be observed not only in FM-coupled but also in AFM-coupled dimers, with a similar magnitude of the energy splitting for both cases. Our theoretical analysis attributes this phenomenon to the breaking of an effective time-reversal symmetry of the AFM dimer, which would otherwise protect the degeneracy of the Shiba states, by SOC in the non-centrosymmetric system. Note that this splitting is not expected to occur for impurities in centrosymmetric bulk systems. There, the atoms in the dimer may be exchanged by spatial inversion *P*, rather than only by the *C*_2_ rotation discussed above. Since the spins remain invariant under spatial inversion, the TP symmetry would then be sufficient to enforce the degeneracy of the Shiba states. An effective time-reversal symmetry *T*_r_ is commonly found not only in antiferromagnetic dimers, but in antiferromagnetic chains and two-dimensional structures as well. The breaking of this symmetry by the SOC in non-centrosymmetric systems should clearly distinguish the emergent MBS in Shiba bands from their counterparts in time-reversal invariant systems^42,47^. Most importantly, our findings indicate that observing the presence or the absence of the splitting of Shiba states in dimers on surfaces cannot be used as a fingerprint for the type of exchange interaction between two magnetic impurities^28,31^. These results should motivate to revisit previous experimental observations of Shiba states and theoretical predictions on the formation of MBS at the ends of Shiba atom chains by taking into account the SOC in systems with broken inversion symmetry^48^, shedding new light on these potential building blocks of topological superconductors.

## Methods

### STM and STS measurements

All experiments were performed in a home-built ultra-high vacuum STM setup, operated at a temperature of 320 mK^49^. STM images were obtained by stabilizing the STM tip at a given bias voltage *V*_bias_ applied to the sample and tunneling current *I*. d*I*/d*V* spectra were obtained using a standard lock-in technique with a modulation frequency of *f*_mod_ = 4142 Hz, a modulation voltage of *V*_mod_ = 20 µV added to *V*_bias_, and stabilization of the tip at *V*_stab_ = 6 mV and *I*_stab_ =1 nA before opening the feedback and sweeping the bias. To visualize spatial distributions of the Shiba states, we defined spatial grids on the structure of interest with a certain pixel resolution, typically with a spacing of about 50–100 pm between grid points. d*I*/d*V* spectra were then measured on every point of the grid, with the parameters listed above except for a different modulation voltage of *V*_mod_ = 40 µV. d*I*/d*V* maps are slices of this d*I*/d*V* grid evaluated at a given bias voltage.

We used an electrochemically etched and in-situ flashed tungsten tip, which was indented a few nanometers into the niobium surface, covering it with niobium and producing a superconducting apex of the tip.

We achieved a considerably better energy resolution as compared to previous results obtained on this sample system^18^ by performing experiments at a lower temperature (320 mK) and making use of superconducting probe tips. Based on a d*I*/d*V* spectrum taken on a patch of clean Nb(110) we find the two substrate coherence peaks at ±2.93 mV as indicated by the red vertical lines in Fig. 1a. From measurements with a normal metal tip, we deduce a superconducting gap of Δ_Nb_ = 1.50 mV (see Supplementary Note 3). Thereby, the shift in the position of the coherence peaks in the measurement with the superconducting STM probe tip can be used to determine its superconducting gap of Δ_tip_ = 1.43 mV ^30^. All Shiba states in the d*I*/d*V* spectra and maps shown here are correspondingly shifted in energy by Δ_tip_.

### Sample preparation

The Nb(110) single crystal with a purity of 99.999% was introduced into the ultra-high vacuum chamber and subsequently cleaned by Ar ion sputtering and flashing to about 2400 °C to remove surface-near oxygen^50^. Mn atoms were evaporated to the sample while keeping the substrate temperature below 10 K, to achieve a statistical distribution of single adatoms (see Fig. 1c, Supplementary Note 1 and Supplementary Fig. 1a). Combining the knowledge of the position of single Mn adatoms with respect to the Nb(110) surface unit cell visible in atomically resolved STM images (Supplementary Fig. 1b) with atom-manipulation images (Supplementary Fig. 1c), with the fact that we find identical features in d*I*/d*V* spectra for all single Mn adatoms, as well as with the results of first-principles calculations (Supplementary Note 6), we conclude that the only energetically stable adsorption site for Mn adatoms on Nb(110) is the hollow site in the center of four Nb atoms (Fig. 1e). Artificial dimers were constructed using STM tip-induced atom manipulation from two single adatoms with a typical tunneling resistance of about 60 kΩ. As shown in atom-manipulation images (Supplementary Fig. 1c), the height of the STM tip reveals a characteristic signal while manipulating an atom from one adsorption site to a neighboring one. This signal is used to predetermine the adsorption sites of the two atoms in the dimer during its manipulation process. In combination with the size and orientation of the elliptical shape revealed in an STM image of the resulting dimer, and the possible adsorption sites given by atom-manipulation images overlaid onto the STM image (Supplementary Note 2 and Supplementary Fig. 2), the positions of the two atoms in each dimer indicated in Figs. 2d and 3c are determined unambiguously.

### Model calculations

The system was described by the Hamiltonian

1 $$H = H_{\mathrm{b}} + H_{\mathrm{R}} + H_{{\mathrm{sc}}} + H_{{\mathrm{imp}}}$$

with the spectrum of bulk Nb

2 $$H_{\mathrm{b}} = \mathop {\sum}\limits_{{\mathbf{k}}} {{{\Phi }}_{{\mathbf{k}}}^\dagger \xi _{{\mathbf{k}}}\tau ^z{{\Phi }}_{\mathbf{k}}}$$

nearest-neighbor Rashba-type SOC on the bcc(110) surface of Nb

3 $$H_{\mathrm{R}} = \mathop {\sum}\limits_{{\mathbf{k}}} {{{\Phi }}_{{\mathbf{k}}}^\dagger } \left[ {4t_{\mathrm{R}}\sin \left( {\frac{{k^x}}{2}a} \right)\cos \left( {\frac{{\sqrt 2 }}{2}k^ya} \right)\sigma _4^y - 4t_{\mathrm{R}}\sin \left( {\frac{{\sqrt 2 }}{2}k^ya} \right)\cos \left( {\frac{{k^x}}{2}a} \right)\sqrt 2 \sigma _4^x} \right]{{\Phi }}_{{\mathbf{k}}}$$

s-wave superconducting pairing

4 $$H_{{\mathrm{sc}}} = \mathop {\sum}\limits_{\mathbf{k}} {{{\Phi }}_{\mathbf{k}}^\dagger } \Delta \tau ^x{{\Phi }}_{\mathbf{k}}$$

and the impurity potential

5 $$H_{{\mathrm{imp}}} = \frac{1}{N}\mathop {\sum}\limits_{\mu ,\alpha ,{\mathbf{k}},{{\mathbf{k}}^\prime } } {{{\Phi }}_{\mathbf{k}}^\dagger } \Psi _\mu \left( {\mathbf{k}} \right)\left( {K^\mu \tau ^z - \frac{{J^\mu S}}{2}S_\mu ^\alpha \sigma _4^\alpha \tau ^z} \right)\Psi _\mu ^ \ast \left( {{\mathbf{k}}^\prime } \right){{\Phi }}_{{{\mathbf{k}}^\prime } }$$

In Eqs. (1)–(5), ${{\Phi }}_{\mathbf{k}} = 1/\sqrt 2 \left( {c_{{\mathbf{k}} \uparrow },c_{{\mathbf{k}} \downarrow },c_{ - {\mathbf{k}} \downarrow }^\dagger ,c_{ - {\mathbf{k}} \uparrow }^\dagger } \right)$ is the Nambu spinor, with the electron annihilation operators $c_{{\mathbf{k}}\sigma }$. The matrices in Nambu space read

6 $$\sigma _4^\alpha = \left[ {\begin{array}{*{20}{c}} {\sigma ^\alpha } & 0 \\ 0 & { - \sigma ^\alpha } \end{array}} \right],\tau ^z = \left[ {\begin{array}{*{20}{c}} {\sigma ^0} & 0 \\ 0 & { - \sigma ^0} \end{array}} \right],\tau ^x = \left[ {\begin{array}{*{20}{c}} 0 & {\sigma ^0} \\ {\sigma ^0} & 0 \end{array}} \right]$$

where *σ*^*α*^ are the usual 2 × 2 Pauli matrices for *α* = *x,y,z* and *σ*^0^ is the unit matrix. The bulk Nb spectrum *ξ*_**k**_ was determined from a Slater–Koster two-center tight-binding model, using the parameters in ref. ^51^. The original spectrum containing 9 bands (5*s*, 5*p*, and 4*d*) was mapped to a single-band model only containing states in the energy range [*E*_F_−10Δ, *E*_F_ + 10Δ]. This was performed to decrease the numerical cost of the wave-vector summations since the contribution of the states far away from the Fermi level to the Shiba states is expected to be small. For a different implementation of the integration in the vicinity of the Fermi surface with an energy cut-off, see ref. ^52^. In the Rashba term, *x* and *y* are along the [001] and $[1\bar 10]$ directions, respectively (Fig. 1e). The Rashba parameter *t*_R_ = 7.5 meV was approximated by identifying Rashba-split surface states in Nb(110) from ab initio calculations based on the screened Korringa–Kohn–Rostoker (SKKR) method (Supplementary Note 7). The order parameter Δ = 1.5 meV was based on the experimentally determined gap size Δ_Nb_ and assumed to be real-valued and homogeneous^17^. In the impurity potential, *S* is the spin quantum number, $\left( {S_\mu ^x,S_\mu ^y,S_\mu ^z} \right)$ is the direction of the localized magnetic moment, and *μ* indexes the different scattering channels, including different orbitals in a single impurity and multiple sites in the case of dimers. $\Psi _\mu \left( {\mathbf{k}} \right) = {\mathrm{diag}}\left( {\psi _\mu \left( {\mathbf{k}} \right),\psi _\mu \left( {\mathbf{k}} \right),\psi _\mu ^ \ast \left( { - {\mathbf{k}}} \right),\psi _\mu ^ \ast \left( { - {\mathbf{k}}} \right)} \right)$ describes the shapes of the scattering centers, with the wave functions *ψ*_*μ*_ expressed in a plane-wave basis. The scattering wave functions were identified with the *d* orbitals of a Mn adatom on Nb(110) calculated using the Vienna Ab-initio Simulation Package (VASP)^53–55^, based on the procedure described in ref. ^20^ (see Supplementary Note 6 for details). The *K*^*μ*^ and *J*^*μ*^*S*/2 parameters were determined by approximating the experimentally observed energy positions and the asymmetries between negative and positive bias for all Shiba states in the adatom. The same values were used for calculating the Shiba states in the dimers. The magnetic and non-magnetic scattering parameters with SOC (used in Figs. 1j–n, 2k–s, and 4f–n) and without it (used in Fig. 4a–e) are listed in Supplementary Tables 1 and 2, respectively. An approximation of the scattering parameters based on the density of states determined from SKKR calculations is discussed in Supplementary Note 8.

The LDOS was calculated using a Green’s function-based method^28,52,56^. The Green’s function at complex energy *z* is expressed as

7 $$G\left( {z,{\mathbf{k}},{{\mathbf{k}}^\prime } } \right) = G_0\left( {z,{\mathbf{k}}} \right)\delta _{{\mathbf{k}},{{\mathbf{k}}^\prime } } + G_0\left( {z,{\mathbf{k}}} \right)T\left( {z,{\mathbf{k}},{{\mathbf{k}}^\prime } } \right)G_0\left( {z,{\mathbf{k}}^\prime} \right)$$

where

8 $$G_0\left( {z,{\mathbf{k}}} \right) = 	\left( z - \xi _{\mathbf{k}}\tau ^z - 4t_{\mathrm{R}}\sin \left( {\frac{{k^x}}{2}a} \right)\cos \left( {\frac{{\sqrt 2 }}{2}k^ya} \right)\sigma _4^y \right. \\ 	\left.+ \,4t_{\mathrm{R}}\sin \left( {\frac{{\sqrt 2 }}{2}k^ya} \right)\cos \left( {\frac{{k^x}}{2}a} \right)\sqrt 2 \sigma _4^x - {{\Delta }}\tau ^x \right)^{ - 1}$$

is the Green’s function of the substrate and

9 $$T\left( {z,{\mathbf{k}},{\mathbf{k}}^\prime } \right) = 	\mathop {\sum}\limits_{\mu ,\mu^\prime } {{{\Psi }}_\mu \left( {\mathbf{k}} \right)} \frac{1}{N}\left( {K^\mu \tau ^z - \frac{{J^\mu S}}{2}S_\mu ^\alpha \sigma _4^\alpha \tau ^z} \right)\\ 	 \times\left[ {I_4 - \mathop {\sum}\limits_{{\mathbf{k}}^{\prime\prime} } {{{\Psi }}_\mu ^ \ast } \left( {{\mathbf{k}}^{\prime\prime} } \right)G_0\left( {z,{\mathbf{k}}^{\prime\prime}e } \right){{\Psi }}_{\mu^\prime }\left( {{\mathbf{k}}^{\prime\prime} } \right)\frac{1}{N}\left( {K^{\mu^\prime }\tau ^z - \frac{{J^{\mu^\prime }S}}{2}S_{\mu^\prime }^\alpha \sigma _4^\alpha \tau ^z} \right)} \right]^{ - 1}{{\Psi }}_{\mu^\prime }^ \ast \left( {{\mathbf{k}}^\prime } \right)$$

is the *T* operator describing scattering of the impurities. *I*_4_ is the unit matrix in Nambu space. Shiba states are found at the real energies $\left| z \right| \, < \,{{\Delta }}$ inside the gap where *T* has poles, meaning that the determinant of the matrix in the brackets equals 0. Finding the zeroes of the determinant made it possible to separate the Shiba states very close in energy in Figs. 2k and 4f, and to confirm the degeneracies for the AFM dimer in the absence of SOC in Fig. 4a.

The LDOS was calculated as

10 $${\mathrm{LDOS}}\,\left( {E,{\mathbf{r}}} \right) = - \frac{1}{\pi }{\mathrm{{ImTr}}}\left[ {\frac{1}{N}\mathop {\sum}\limits_{{\mathbf{k}},{\mathbf{k}}^\prime } {e^{ - {\mathrm{i}}\left( {{\mathbf{k}} - {\mathbf{k}}^\prime } \right){\mathbf{r}}}} G\left( {E + {\mathrm{i}}\delta ,{\mathbf{k}},{\mathbf{k}}^\prime } \right)\frac{{I_4 + \tau ^z}}{2}} \right]$$

after Fourier transformation back into real space and projecting the spectral function on the particle part of Nambu space. In Eq. (10), δ denotes a small imaginary part, which was set to $k_{\mathrm{B}} \cdot 300\,{\mathrm{mK}}$ to approximate the experimentally observed energy resolution. The spectra in Figs. 1j, 2k, 4a and f were obtained by calculating a weighted average of the LDOS over different positions,

11 $${\mathrm{LDOS}}_{{\mathrm{spectra}}}\left( E \right) = \mathop {\sum}\limits_{i = 1}^9 {e^{ - \frac{{r_i}}{a}}} {\mathrm{LDOS}}\left( {E,{\mathbf{r}}_i} \right)$$

with ${\mathbf{r}}_i = a\left( {{\mathrm{cos}}\frac{{i\pi }}{4},{\mathrm{sin}}\frac{{i\pi }}{4},0} \right)$ for $i = 1, \ldots ,8$ and ${\mathbf{r}}_9 = \left( {0,0,0} \right)$, measured with respect to the position of the single atom or with respect to the position halfway between the two atoms forming the dimer, respectively. The averaging was performed since some of the orbitals have a very small spectral weight when measured directly on the adatom due to their symmetry.

## Supplementary information

Supplementary Information

## Data Availability

The authors declare that the data supporting the findings of this study are available within the paper and its supplementary information files.

## Code availability

The analysis codes that support the findings of the study are available from the corresponding authors on reasonable request.
