# Supplementary material for: Spin-orbit coupling induced splitting of Yu-Shiba-Rusinov states in antiferromagnetic dimers
Source: Nat Commun. 2021 Apr 1;12:2040. doi: 10.1038/s41467-021-22261-6 (PMC8016932; doi:10.1038/s41467-021-22261-6)
Supplement: Supplementary file 1 — Supplementary Information [file 41467_2021_22261_MOESM1_ESM.pdf]

Supplementary Information

Spin-orbit coupling induced splitting of Yu-Shiba-Rusinov states in antiferromagnetic dimers

Philip Beck[1], Lucas Schneider[1], Levente Rózsa[2†], Krisztián Palotás[3,4,5], András Lászlóffy[3,5], László Szunyogh[5,6], Jens Wiebe[1*] and Roland Wiesendanger[1]

[1] Department of Physics, University of Hamburg, D-20355 Hamburg, Germany

[2] Department of Physics, University of Konstanz, D-78457 Konstanz, Germany

[3] Institute for Solid State Physics and Optics, Wigner Research Center for Physics, H-1525 Budapest, Hungary

[4] MTA-SZTE Reaction Kinetics and Surface Chemistry Research Group, University of Szeged, H-6720 Szeged, Hungary

[5] Department of Theoretical Physics, Budapest University of Technology and Economics, H-1111 Budapest, Hungary

[6] MTA-BME Condensed Matter Research Group, Budapest University of Technology and Economics, H-1111 Budapest, Hungary

Contact information: *jwiebe@physnet.uni-hamburg.de, †levente.rozsa@uni-konstanz.de

Supplementary Note 1. Sample preparation

An overview image of the sample surface, which was used for the experiments, is shown in Supplementary Fig. 1a. Using the procedure described in the Methods section of the main text, we obtained a similar Nb(110) surface quality as previously reported in the literature[1]. Only a few dark depressions, which can be identified as oxygen impurities, remain on the surface. Clean patches of Nb(110), at least 5 nm x 5 nm in size, could easily be found and were used for the scanning tunneling spectroscopy (STS) experiments in order to avoid any influence of the oxygen impurities. Supplementary Fig. 1b shows an atomically resolved STM image. White lines tracing the rows of surface Nb atoms along the two crystallographic directions are added as a guide to the eye. We find that the centers of the Mn adatoms (bright protrusions) lie in the rectangular area defined by two neighboring lines along the $[001]$ and two neighboring lines along the $[1\bar{1}0]$ direction. An atom-manipulation image[2], which is obtained by pushing or dragging a single Mn adatom across the sample surface while recording an STM image at high tunneling conductance, is shown in Supplementary Fig. 1c. We find that the symmetry and the distances of the protrusions in the manipulation image follow that of the four-fold coordinated hollow sites of the Nb(110) surface. In combination with the information drawn from Supplementary Fig. 1b and VASP calculations (Supplementary Note 6), we conclude that this hollow site, for which a Mn adatom adsorbs in the diamond-shaped center of four surface Nb atoms, is the only favorable and observable adsorption site.

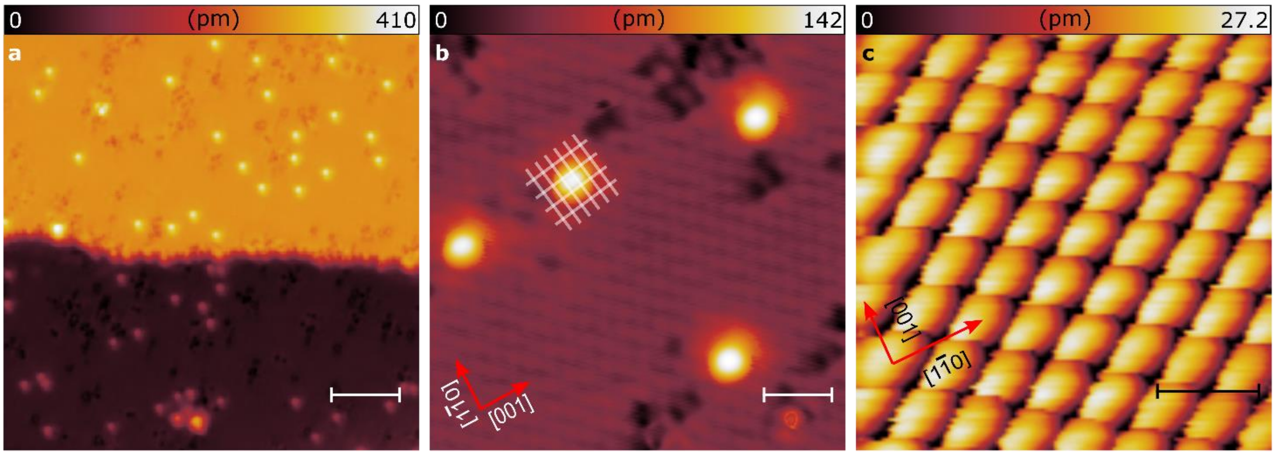

Supplementary Figure 1 | Overview of Mn/Nb(110) sample and Mn adsorption site. a, Large-scale STM image providing an overview of the prepared Mn/Nb(110) sample. A small amount of oxygen contaminations remained on the sample, which are visible as dark depressions. The white scale bar represents a length of 5 nm (V_{bias}=6 mV and I=200 pA). **b**, Atomically resolved STM image of the Nb(110) surface including four single Mn adatoms in the scan frame. White lines trace the rows of surface Nb atoms along the $[001]$- and $[1\bar{1}0]$-directions. The white scale bar represents a length of 2 nm (V_{bias}=6 mV and I=1 nA). **c**, Atom-manipulation image obtained by dragging/pushing a Mn adatom across the Nb(110) surface while taking the image. The black scale bar represents a length of 500 pm (V_{bias}=-3 mV and I=50 nA).

Supplementary Note 2. Determining the positions of the two atoms in the dimers

Supplementary Fig. 2 shows STM images and height profiles across a single Mn atom and close-packed dimers which have been assembled by STM tip-induced atom manipulation in different crystallographic directions as described in the Methods section of the main text. In comparison to the Mn atom, which has an almost circular shape, each Mn dimer has an elliptical shape. The ellipse is oriented along the specific crystallographic direction which coincides with the orientation of the two atoms in the dimer predetermined by the manipulation process. In addition, atom-manipulation images taken on the bare Nb substrate overlaid on these STM images reveal the possible adsorption positions of the two atoms in the dimer. Combining the lattice positions predetermined by the atom manipulation process, the orientation and size of the elliptical shape of the dimer from the STM image, and the possible adsorption sites from the overlaid atom manipulation image, we can unambiguously determine the positions of the two atoms in

each of the manipulated dimers. These positions are indicated in Supplementary Fig. 2 by red circles as well as in Figs. 2c,d and Figs. 3b,c. Finally, as revealed by the line profiles in Supplementary Fig. 2, the dimers have an increased apparent height compared to the single Mn adatom.

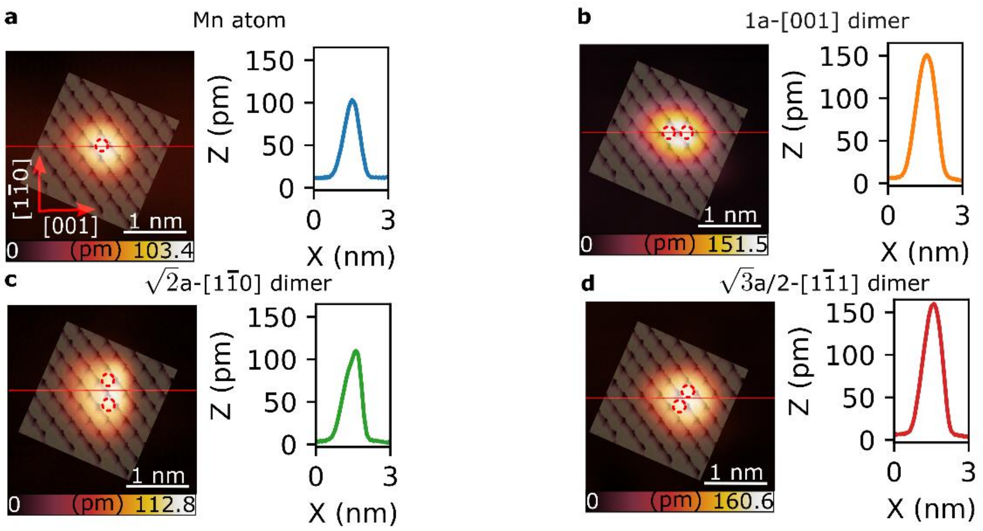

Supplementary Figure 2 | Positions of the two atoms in the manipulated dimers. a-d, STM images taken using the same tip with overlaid atom-manipulation images (left panels) and height profiles along the indicated red lines (right panels) of a Mn atom (**a**), a $1\mathrm{a}-[001]$ dimer (**b**), a $\sqrt{2}\mathrm{a}-[1\bar{1}0]$ dimer (**c**), and a $\sqrt{3}\mathrm{a}/2-[1\bar{1}1]$ dimer (**d**). The determined positions of the two atoms in each dimer are indicated by red circles. The crystallographic orientation of all images is indicated by the vectors in **a**. (STM images: V_{bias}=6 mV and I=1 nA; atom manipulation images: V_{bias}=-3 mV and I=50 nA).

Supplementary Note 3. Determining the tip gap

Accurately knowing the size of the superconducting gap of the STM tip is important if one wants to determine the exact energetic positions of the Shiba states, since all features are shifted by $\pm\Delta_{\mathrm{tip}}$ to higher bias voltages. One way to characterize the superconducting gap of the tip is based on a comparison with STS measurements using a normal conducting metal tip. An example of a tunneling spectrum obtained with a normal conducting Cr tip on a Nb(110) sample is shown in Supplementary Fig. 3a. The spectrum is fitted by standard STS theory using a Dynes function in order to model the substrate's local density of states (LDOS)

$$\rho(E = eV) = \rho_0 \cdot \mathrm{Re}\left(\frac{E - \mathrm{i}\gamma}{\sqrt{(E - \mathrm{i}\gamma)^2 - \Delta^2}}\right). \quad (1)$$

The resulting superconducting gap of Nb is Δ_{Nb}=1.50 mV, which is indicated by the red vertical lines. Since we now know the actual superconducting gap Δ_{Nb} of the sample measured at 320 mK, we can calculate the superconducting gap Δ_{tip} of the tip used in all other measurements. We recall the positions of the coherence peaks indicated in Fig. 1a of the main text (±2.93 mV) which are given by ±(Δ_{Nb}+Δ_{tip}). By a simple subtraction of the fitted gap of Nb (Δ_{Nb}) from this quantity, we obtain a tip gap of $\Delta_{\mathrm{tip}} = 1.43\ \mathrm{mV}$. This value is substantiated by a corresponding fit of a substrate spectrum taken with the superconducting tip assuming two Dynes functions (Supplementary Eq. (1)), one for the substrate's LDOS for which we now know the fitting parameters, which are then used to calculate a convolution matrix (see ref.[3]), and one for the tip's DOS with a different set of parameters, still to be optimized. The multiplication of the convolution matrix with the tip DOS is fitted to the d*I*/d*V* spectrum measured on bare Nb(110) which is shown in Supplementary Fig. 3b.

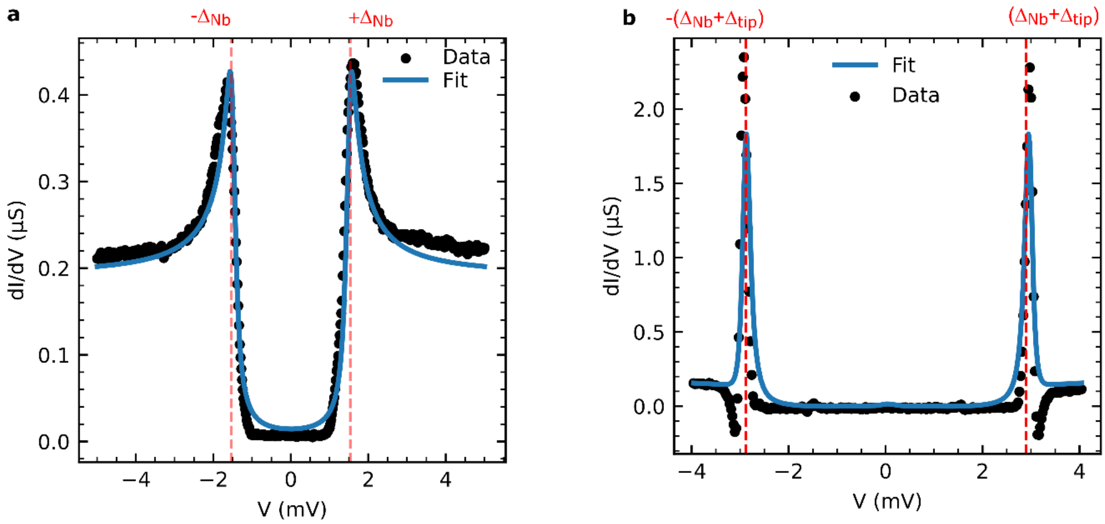

Supplementary Figure 3 | Determination of the substrate and tip gaps. a, d*I*/d*V* spectrum (black dots) taken on bare Nb(110), using a non-superconducting Cr tip. The spectrum was fitted (blue curve) assuming a broadened Dynes function, Supplementary Eq. (1), as the LDOS of the superconducting Nb(110). The extracted fitting parameters are $\Delta_{\mathrm{Nb}} = 1.50\ \mathrm{mV}$ and $\gamma = 0.12\ \mathrm{mV}$. Red vertical lines indicate the position of $\pm\Delta_{\mathrm{Nb}}$ (V_{stab}=-5 mV, I_{stab}=1 nA, V_{mod}=40 µV, Cr tip). **b,** d*I*/d*V* spectrum (black dots) taken on bare Nb(110), using the superconducting tip, which was used for all measurements discussed in the main text. The spectrum was fitted (blue curve) assuming two broadened Dynes functions, one for the substrate's LDOS with fixed parameters (see **a**) and one for the tip's DOS (Supplementary Eq. (1)). The extracted fitting parameters are $\Delta_{\mathrm{tip}} = 1.43\ \mathrm{mV}$ and $\gamma = 300\ \mathrm{nV}$. Red vertical lines indicate the positions of $\pm(\Delta_{\mathrm{Nb}} + \Delta_{\mathrm{tip}})$ (V_{stab}=6 mV, I_{stab}=1 nA, V_{mod}=40 µV, Nb-coated W tip).

Supplementary Note 4. Spin-polarized STM measurements

A recent publication on spin-polarized STM (SP-STM) of Mn chains assembled atom-by-atom using tip-induced atom manipulation on clean Nb(110) discusses the experimentally observed magnetic coupling for a chain of Mn adatoms spaced by $\sqrt{3}a/2$ and built along the $[1\bar{1}1]$-direction and for a chain of Mn adatoms spaced by 2a built along the $[001]$-direction[4]. In both of these chains the antiferromagnetic state observed in a small magnetic field indicates that the neighboring Mn adatoms are AFM coupled.

To demonstrate the FM coupling of Mn adatoms with a spacing of $\sqrt{2}$a along the $[1\bar{1}0]$-direction, the results of an SP-STM measurement using Cr bulk tips on a chain of 11 Mn adatoms with nearest-neighbor separation of $\sqrt{2}$a along the $[1\bar{1}0]$-direction are shown in Supplementary Fig. 4. The same microtip and measurement conditions as in ref.[4] were used for this measurement. An STM image of the Mn_{11} chain is shown in Supplementary Fig. 4a. d*I*/d*V* maps taken on the same area across this chain in +0.5 T and -0.5 T magnetic field, i.e. pointed along opposite directions, are shown in Supplementary Figs. 4b and c, respectively. The contrast of the color scale is adjusted to the same values for both images. One finds that the Nb(110) substrate has the same color in both magnetic fields (compare edges of Supplementary Figs. 4b and c). However, on top of the chain one finds different signals due to the tunneling magnetoresistance. In a magnetic field of +0.5 T, the d*I*/d*V* signal on the chain is lower than it is in a magnetic field of -0.5 T, indicating that the chain's overall magnetization follows the external magnetic field and is aligned parallel and antiparallel to the stable Cr tip magnetization. Since the d*I*/d*V* signal is constant along the chain for each magnetic field, we conclude that the chain is in a ferromagnetically ordered state.

The asymmetry in the measured differential conductance obtained from the d*I*/d*V* maps in opposite magnetic fields is given by:

$$A_{\mathrm{SP}} = \frac{\mathrm{d}I/\mathrm{d}V(V,\mathbf{r}_{\mathrm{t}})|_{-0.5\mathrm{T}} - \mathrm{d}I/\mathrm{d}V(V,\mathbf{r}_{\mathrm{t}})|_{+0.5\mathrm{T}}}{\mathrm{d}I/\mathrm{d}V(V,\mathbf{r}_{\mathrm{t}})|_{-0.5\mathrm{T}} + \mathrm{d}I/\mathrm{d}V(V,\boldsymbol{r}_{\mathrm{t}})|_{+0.5\mathrm{T}}}, \quad (2)$$

where V is the tunneling bias voltage, and $\mathbf{r}_{\mathrm{t}}$ is the position of the tip apex[5]. A map of this asymmetry calculated from Supplementary Figs. 4b and c is shown in Supplementary Fig. 4d and clearly shows FM order. Therefore, we conclude that Mn adatoms with a spacing of $\sqrt{2}$a along the $[1\bar{1}0]$-direction are FM coupled.

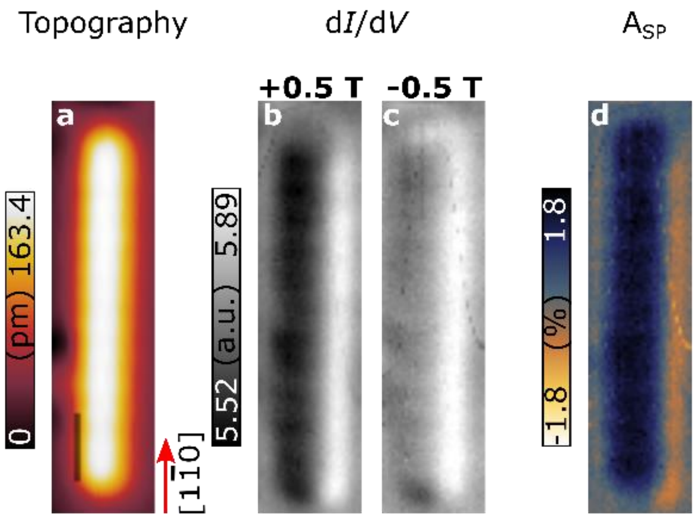

Supplementary Figure 4 | Magnetic structure of a chain of 11 Mn adatoms with a spacing of $\sqrt{2}$a along the $[1\bar{1}0]$-direction. **a**, STM image of the Mn_{11} chain, which was constructed using atom manipulation. **b** and **c**, are the corresponding d*I*/d*V* maps measured with a Cr bulk tip at opposite magnetic fields of +0.5 T and -0.5 T (V_{bias}=6 mV, *I*=1 nA, V_{mod}=2 mV). Both maps are adjusted to the same color scale for comparability. **d**, Asymmetry (A_{SP}) map calculated from the d*I*/d*V* maps in opposite magnetic fields (**b**, **c**) according to Supplementary Eq. (2). The black scale bar in **a** represents a length of 1 nm and is valid for all panels.

Supplementary Note 5. Complementary spectroscopic data

The following figures, Supplementary Figs. 5a-d, Supplementary Fig. 6 and Supplementary Fig. 7 contain the positive bias counterparts of the d*I*/d*V* maps shown in the main text of a single adatom (Figs. 1f-i), a $\sqrt{2}\mathrm{a} - [1\bar{1}0]$ dimer (Figs. 2e-j) and a $\sqrt{3}\mathrm{a}/2 - [1\bar{1}1]$ dimer (Figs. 3d-i), respectively. They allow for the same conclusions regarding the hybridization of Shiba states which were drawn in the main text and support these findings.

Additionally, Supplementary Figs. 5e-l show larger scale (5 nm x 5 nm) d*I*/d*V* maps measured on a single Mn adatom, to display the strong spatial decay of the spectral intensity of the Shiba states. To make the long-range extension visible at all, the stabilization current was increased to $I_{\mathrm{stab}} = 3$ nA, compared to $I_{\mathrm{stab}} = 1$ nA which was used for all other d*I*/d*V* maps. Furthermore, the contrast had to be adjusted.

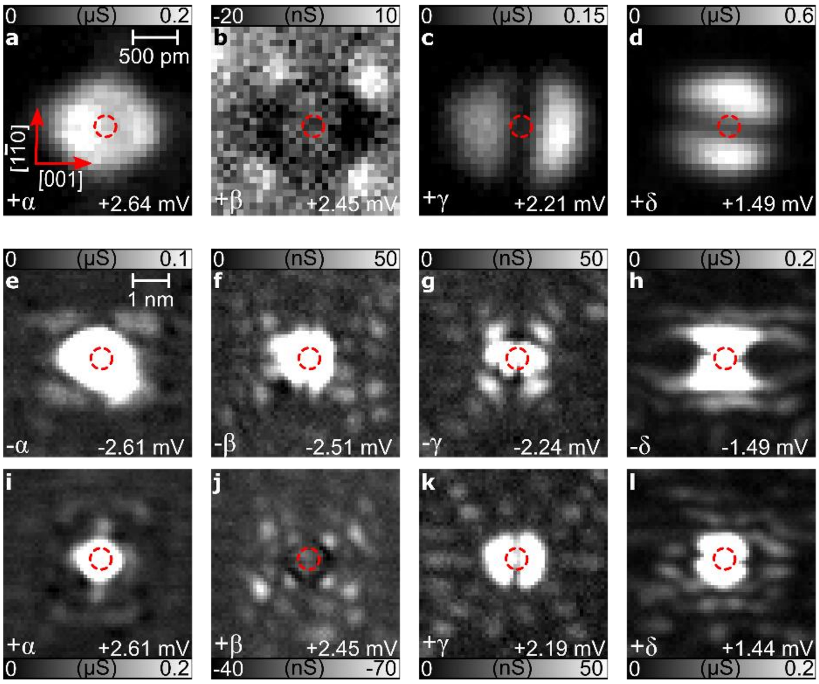

Supplementary Figure 5 | Positive bias and large-scale d*I*/d*V* maps of Shiba states of a single Mn adatom. a-d, Positive bias counterpart of the d*I*/d*V* maps shown in Figs. 1f-i in the main text of a single Mn adatom taken at the bias voltages as indicated (V_{stab}=6 mV, I_{stab}=1 nA, V_{mod}=40 µV). Red circles denote the position of the adatom. **e-l**, Large-scale d*I*/d*V* maps ($5\ \mathrm{nm} \times 5\ \mathrm{nm}$), recorded with bias voltages as indicated and obtained with a lower stabilization height compared to all other d*I*/d*V* maps shown in this work. The color maps are adjusted to lower d*I*/d*V* values in order to enhance the visibility of the long-range extension of the Shiba states (V_{stab}=6 mV, I_{stab}=3 nA, V_{mod}=40 µV).

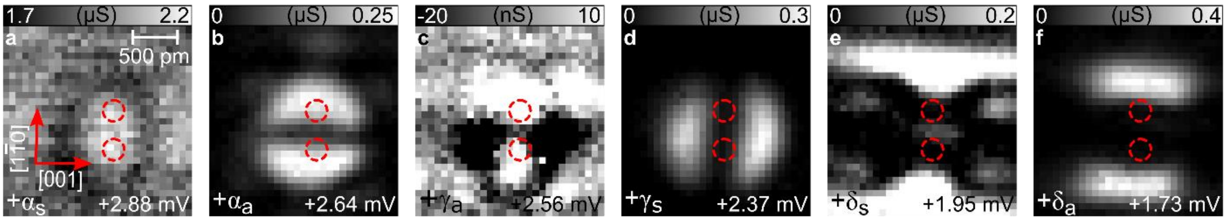

Supplementary Figure 6 | Hybridization of Shiba states in the FM-coupled $\sqrt{2}\mathbf{a}-[\mathbf{1\bar{1}0}]$ Mn dimer. a-f, Positive bias counterpart of the d*I*/d*V* maps shown in Figs. 2e-j of the main text for the $\sqrt{2}\mathrm{a}-[1\bar{1}0]$ Mn dimer at the respective bias voltages, where Shiba states were identified (V_{stab}=6 mV, I_{stab}=1 nA, V_{mod}=40 µV). Red circles denote the positions of the Mn adatoms in the dimer.

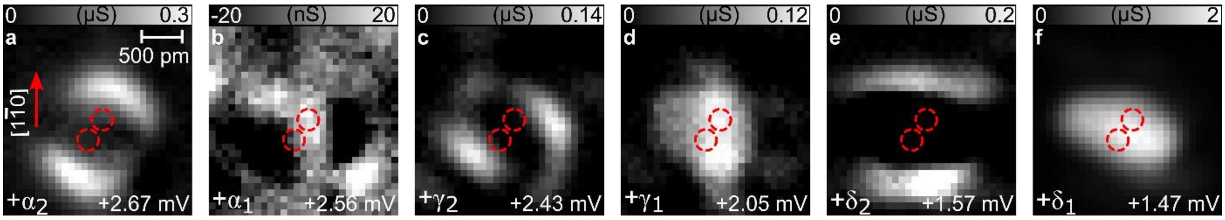

Supplementary Figure 7 | Hybridization of Shiba states in the AFM-coupled $\sqrt{3}\mathrm{a}/2-[1\bar{1}1]$ Mn dimer. a-f, Positive bias counterpart of the dI/dV maps shown in Figs. 3d-i of the main text for the $\sqrt{3}\mathrm{a}/2-[1\bar{1}1]$ Mn dimer at the respective bias voltages, where Shiba states were identified (V_{stab}=6 mV, I_{stab}=1 nA, V_{mod}=40 μV). Red circles denote the positions of the Mn adatoms in the dimer.

In Supplementary Fig. 8 we present another example of split Shiba states in an AFM-coupled dimer, i.e., the $2\mathrm{a}-[001]$ Mn dimer. The AFM coupling of two atoms with that distance is concluded from SP-STM measurements of chains along this direction with the same nearest-neighbor separation[4] (Supplementary Note 4) and from our calculations based on the KKR method which reveal an AFM ground state for the dimer (Supplementary Table 3). A dI/dV spectrum taken on a single Mn adatom and one taken on the $2\mathrm{a}-[001]$ dimer is shown in Supplementary Fig. 8a. A small-scale STM image of the dimer is shown in Supplementary Fig. 8b. dI/dV maps, taken at all bias voltages where a peak can be identified in Supplementary Fig. 8a are shown in Supplementary Figs. 8c-l. The spatial distributions of the Shiba states in Supplementary Figs. 8c and d resemble that of two $\pm\alpha$ states of two Mn adatoms which are not split into hybridized combinations. However, similar to the case of the $\sqrt{3}\mathrm{a}/2-[1\bar{1}1]$ Mn dimer shown in Fig. 3 of the main text, we find that the $\pm\gamma$ and the $\pm\delta$ states are split into pairs of hybridized combinations of the single-adatom Shiba states also for this AFM-coupled dimer (Supplementary Figs. 8e-l). We correspondingly name them $\pm\gamma_1$ and $\pm\gamma_2$, and $\pm\delta_1$ and $\pm\delta_2$. While the $+\gamma_2$ and $+\delta_2$ states have clear nodal lines between the two adatoms (Supplementary Figs. 8f and l), the $-\gamma_1$ and $-\delta_1$ states reveal maxima in intensity at the corresponding locations (Supplementary Figs. 8g and i). Note that this classification into antisymmetric and symmetric hybridizations of the corresponding single-adatom Shiba states is not as obvious for some of the particle-hole partners of these states (see the $+\gamma_1$ state in Supplementary Fig. 8h and the $+\delta_1$ state in Supplementary Fig. 8j). Instead, we can conclude that overall, the split Shiba states at positive energy appear rather antisymmetric compared to their negative-energy partners which are rather symmetric.

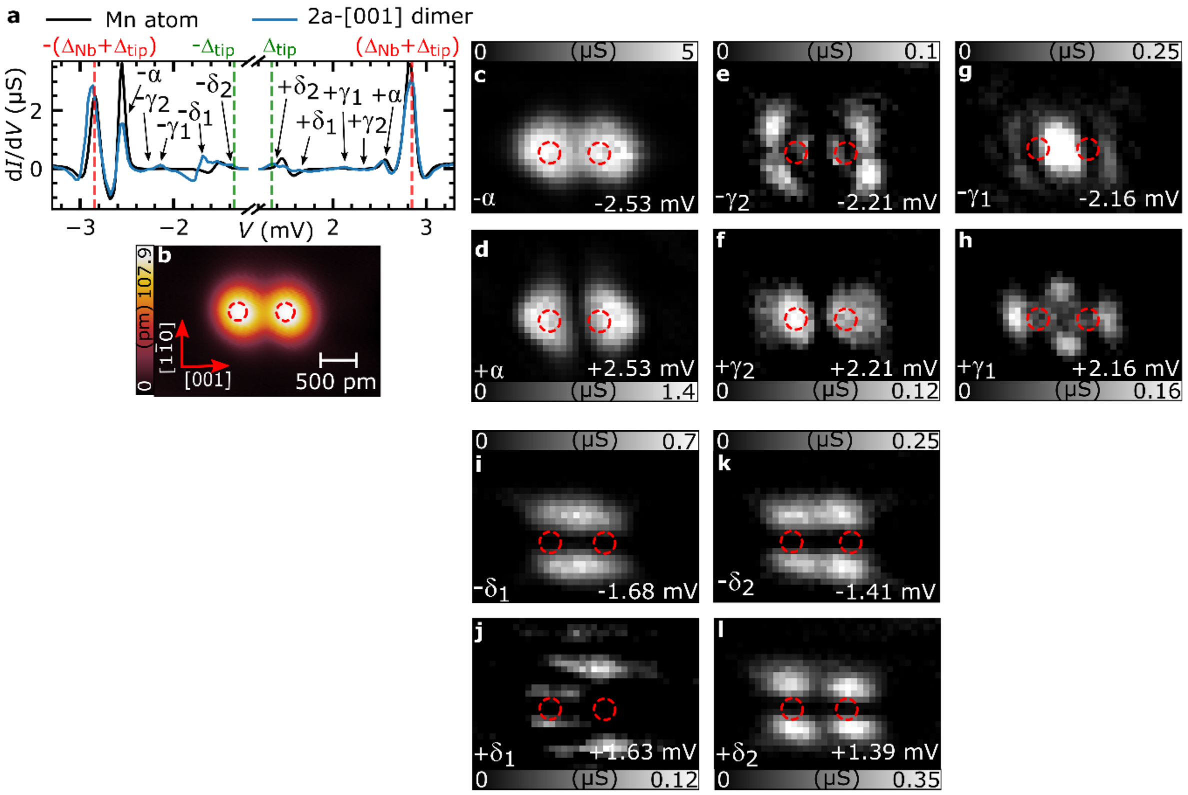

Supplementary Figure 8 | STS study of hybridized Shiba states of an AFM-coupled $2a-[001]$ Mn dimer. a, d*I*/d*V* spectra obtained in the middle of the two adatoms forming the $2a-[001]$ Mn dimer (blue) and a reference spectrum taken on a single adatom (black). The superconducting energy gap of the tip is smaller in these spectra ($\Delta_{\mathrm{tip}} = 1.37\ \mathrm{mV}$) compared to all other single spectra and d*I*/d*V* maps displayed in this work. **b**, STM image of the $2a-[001]$ dimer (V_{bias}=-6 mV and I=1 nA). The red arrows indicate the crystallographic directions of the Nb(110) surface. **c-l,** d*I*/d*V* maps displaying the spatial distributions of the five pairs of Shiba states marked in **a**. The lattice vectors and the lateral scale bar marked in **b** are valid for all the maps. (V_{stab}=-6 mV, I_{stab}=1 nA, V_{mod}=20 μV).

Supplementary Note 6. VASP calculations

The equilibrium geometry of the Mn adatom on Nb(110) and the shapes of the scattering potentials were determined using the Vienna Ab-initio Simulation Package (VASP)[6–8]. The considered supercell included a single Mn adatom deposited in a hollow position on 4 layers of Nb, with 7 × 7 atoms in each layer in the bcc(110) geometry, using the bulk lattice constant $a = 330.04\ \mathrm{pm}$. A sufficiently thick vacuum region of close to 10 Å was considered in the direction perpendicular to the surface to minimize interactions between repeated supercells. Exchange–correlation effects were included using the potential construction by Perdew, Burke and Ernzerhof[9], and reciprocal-space calculations were

restricted to the Γ point due to the large size of the supercell. The Mn adatom and the atoms in the top Nb layer were allowed to relax vertically. It was found that the average vertical distance between the atoms in the two highest Nb layers decreased to $227.49\ \mathrm{pm}$ from the bulk value of $\frac{\sqrt{2}}{2}\mathrm{a} = 233.37\ \mathrm{pm}$, and that the Mn adatom relaxed even further, to a vertical distance of $198.68\ \mathrm{pm}$ measured from its nearest Nb neighbours. The spin magnetic moment of the Mn adatom was found to be 3.60 μ_B. It was confirmed that the total energy in the hollow adsorption site was about 580 meV lower than on top of the Nb atoms, and that the adatom relaxed back into the hollow position when starting the relaxation with a slightly horizontally displaced position. This confirms the hollow position as the only energetically favorable adsorption site for the Mn adatom on the surface.

The scattering wave functions ψ_μ indirectly entering Eq. (5) of the main text were extracted from the calculations similar to the procedure discussed in ref.[10]. Using the self-consistently optimized geometry, VASP calculations were performed for a minimal cluster consisting of the Mn adatom and its four nearest-neighbor Nb atoms, sufficient for reproducing the crystal-field splitting of the orbitals in the $C_{2\mathrm{v}}$ point group. The distance between the Mn adatoms in repeated supercells was at least 20 Å, sufficiently large to avoid interactions between the periodic images, and a $3 \times 3 \times 1$ $\mathbf{k}$ mesh was used.

By inspecting the orbital-resolved weights of the wave functions projected on the Mn adatom, it was possible to identify the five occupied 3*d* states in the majority spin channel, as expected for Mn with a half-filled *d* band. In the minimal cluster, these states are still strongly localized on the Mn adatom. There was a single state for each of the d_{xy}, d_{yz} and d_{xz} orbitals, and two of them were a linear combination of the hybridized d_{z^2} and $d_{x^2-y^2}$ states, as expected based on symmetry arguments (see Supplementary Note 10).

The wave-function coefficients for the identified states were extracted using the WaveTrans program[11]. The reciprocal space coefficients were transformed to real space[12] in a volume centered on the adatom and being similar to the VASP supercell in size. Then, the $\psi_\mu(\mathbf{k})$ coefficients were determined on the finer grid in the vicinity of the Fermi level where the spectrum $\xi_\mathbf{k}$ was calculated, following the energy cut-off discussed in the Methods section of the main text. Due to the fine grid in reciprocal space, the artificial periodicity of the wave function introduced by the supercell approach is suppressed. The wave vectors were restricted to the first Brillouin zone, avoiding periodicity in reciprocal space, thereby obtaining a smooth wave function in real space rather than peaks localized at the real-space lattice sites. The

latter property allows for a finer spatial resolution of the Shiba states compared to what can be achieved in lattice tight-binding models, which was necessary because the experimentally observed extension of the Shiba states is restricted to a few lattice constants.

The non-magnetic K^{μ} and magnetic $J^{\mu}S/2$ scattering parameters were determined from a comparison of the calculated LDOS at the adatom with the experimental spectra, as mentioned in the main text. Four different Shiba states were identified experimentally, each of them belonging to a different irreducible representation of the $C_{2\mathrm{v}}$ point group. Therefore, we set the scattering parameters for the wave function with the strongest $d_{x^2-y^2}$ character to zero, so that it does not produce a Shiba state. The K^{μ} and $J^{\mu}S/2$ parameters for the remaining four scattering channels are listed in Supplementary Tables 1 and 2 with and without spin–orbit coupling, respectively. Note that turning the spin–orbit coupling on or off influences the density of states at the Fermi level, thereby requiring a different set of parameters to describe the same energy positions of the Shiba states.

state	K^{μ} (eV)	$J^{\mu}S/2$ (eV)
$d_{z^2} = \alpha$	0.0360	0.0610
$d_{xy} = \beta$	-0.0010	0.1070
$d_{xz} = \gamma$	0.0220	0.0980
$d_{yz} = \delta$	-0.0090	0.0450

Supplementary Table 1 | Scattering parameters for the different scattering channels with spin–orbit coupling. These values were used for the calculations shown in Figs. 1j-n, 2k-s and 4f-n of the main text.

state	K^{μ} (eV)	$J^{\mu}S/2$ (eV)
$d_{z^2} = \alpha$	0.0180	0.0330
$d_{xy} = \beta$	0.0040	0.0780
$d_{xz} = \gamma$	0.0300	0.0750
$d_{yz} = \delta$	-0.0060	0.0335

Supplementary Table 2 | Scattering parameters for the different scattering channels without spin–orbit coupling. These values were used for the calculations shown in Figs. 4a-e of the main text.

Supplementary Note 7. Determining the Rashba parameter based on SKKR calculations

Using the optimized geometry of the surface layer and the adsorbed adatom obtained from VASP calculations (Supplementary Note 6), the surface electronic structure and the magnetic properties of the Mn adatoms and dimers were investigated in more detail based on the fully relativistic screened Korringa–Kohn–Rostoker (SKKR) method[13]. After performing self-consistent calculations for bulk Nb, an interface consisting of 8 Nb layers and 4 layers of empty spheres along the (110) direction was treated. The self-consistent calculations were performed with 16 energy points on a semicircle contour, using 253 $\mathbf{k}$ points in the irreducible part of the surface Brillouin zone and an angular momentum cutoff of $l_{\max} = 3$.

The Rashba parameter in Eq. (3) of the main text was determined by calculating the Bloch spectral function in the surface Nb layer, defined as[14,15]

$$A_L^{\mathrm{B}}(E, \mathbf{k}_{\parallel}) = -\frac{1}{\pi} \mathrm{ImTr} \int G_{LL}^{+}(E, \mathbf{k}_{\parallel}, \mathbf{r}) \mathrm{d}^3\mathbf{r}, \quad (3)$$

where E is the energy, $\mathbf{k}_{\parallel}$ is the in-plane wave vector, L is the layer index, and the trace is taken in angular momentum space. $A_L^{\mathrm{B}}(E, \mathbf{k}_{\parallel})$ corresponds to the density of states resolved in energy and in wave vector, where integration of the retarded Green's function G_{LL}^{+} is only carried out in real space over the atomic sphere.

The Bloch spectral function along the high-symmetry lines in the surface Brillouin zone is displayed in Supplementary Fig. 9. The hexagonal Brillouin zone of the bcc(110) surface is shown in a sketch in the figure. Sharp peaks in the Bloch spectral function in the energy-wave vector plane localized in the few top layers of the Nb(110) surface correspond to surface states, while bulk states appear as a smooth background since the translational symmetry perpendicular to the surface is broken, effectively corresponding to an integration along the k^z component of the wave vector. Surface states may be observed in regions in energy and wave vector space where there are no states in the bulk, for example in the region highlighted by the black ellipse along the $\overline{\Gamma \mathrm{P}}$ line (see also ref.[1]). The spin–orbit coupling causes a clearly identifiable Rashba splitting in these surface states. It was confirmed that these resonances disappear in the lower Nb layers, and the splitting is no longer observable if spin–orbit coupling is turned off in the calculations. The Rashba parameter was extracted by calculating the energy splitting of the peaks ΔE in the surface states, and comparing this value to the expression derived from Eq. (3) in the main text,

$$\Delta E = 2\sqrt{\left[4t_{\mathrm{R}}\sin\left(\frac{k^x}{2}a\right)\cos\left(\frac{\sqrt{2}}{2}k^y a\right)\right]^2 + \left[4\sqrt{2}t_{\mathrm{R}}\sin\left(\frac{\sqrt{2}}{2}k^y a\right)\cos\left(\frac{k^x}{2}a\right)\right]^2} \tag{4}$$

It was found that t_{R}=7.5 meV provides a reasonable agreement with the *ab initio* data in the whole highlighted range, and this value was used in turn for the Shiba state calculations in Figs. 1, 2 and 4 in the main text.

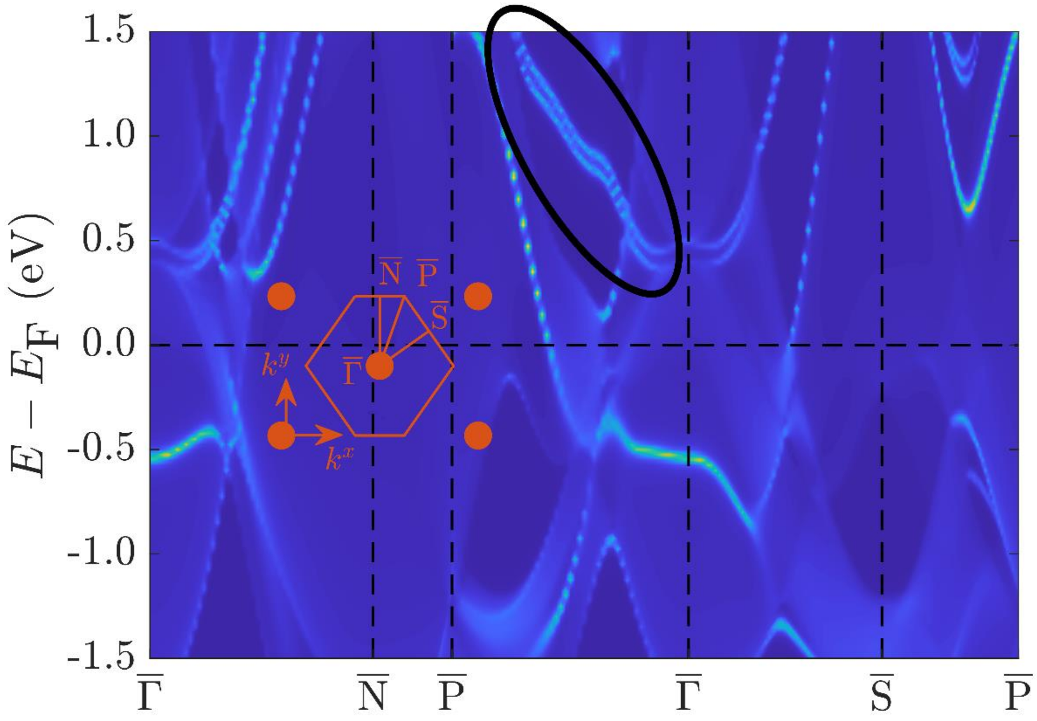

Supplementary Figure 9 | Surface electronic structure of Nb(110). Brillouin spectral function from Supplementary Eq. (3) calculated along the high-symmetry directions of the Brillouin zone of the Nb(110) surface using the SKKR method. The surface Brillouin zone is illustrated in the sketch in the figure. The black ellipse highlights the surface states used to determine the Rashba parameter. Dashed black lines are guides to the eye.

Supplementary Note 8. Approximation of the scattering potential strength based on SKKR calculations

Instead of determining the K^{μ} and $J^{\mu}S/2$ parameters by comparison with the experimental results as described in Supplementary Note 6, they may also be approximated based on the on-site energies and spin splittings of the energy levels of the adatom, along with the strength of the hybridization between the adatom and the substrate, as discussed in refs.[10,16]. These parameters are accessible in first-principles calculations, for example by calculating the density of states at the adatom position.

SKKR calculations based on the embedded cluster algorithm[17] were performed by introducing the Mn adatom in the first vacuum layer of the Nb(110) surface calculated self-consistently in Supplementary Note 7. In the cluster, the 4 Nb atoms were included closest to the Mn adatom in the layer below, as well as 10 empty spheres from the same layer and the layer above. The spin magnetic moment of the adatom was found to be 3.70 μ_B, in good agreement with the VASP calculations in Supplementary Note 6. The magnetic easy axis of the adatom was found to lie along the out-of-plane direction, preferred by 0.15 meV over the $[001]$ and by 0.27 meV over the $[1\bar{1}0]$ direction, as determined from rotating the magnetic moments in the whole cluster and calculating the band energy based on Lloyd's formula[18]. This supports the out-of-plane magnetic alignment of the adatom and the dimers, assumed in the main text. It was confirmed that these values changed by less than 1% when increasing the cluster size up to 291 atoms.

The DOS on the Mn adatom and in the top Nb layer without the adatom were calculated fixing the imaginary part to 68 meV and changing the energy along the real axis. They are shown in Supplementary Figs. 10a and b, respectively. Spin–orbit coupling was turned off in these calculations, such that the eigenstates may be characterized by spin and angular momentum quantum numbers. Only the projections on the real $l = 2$ spherical harmonics, or *d* states, are displayed in Supplementary Fig. 10. One or more resonance peaks may be observed on the adatom in each orbital channel, indicating that the atomic orbitals of Mn hybridize with the metallic Nb substrate and split in energy due to the crystal field. All resonance peaks are located below the Fermi level in the majority spin channel and above it in the minority channel, as expected for Mn with a half-filled *d* band. The hybridization strength depends on the orbitals. For example it is notably weak for the d_{xy} and $d_{x^2-y^2}$ orbitals which overlap less with the Nb atoms in the layer below, leading to sharp resonance peaks. Multiple peaks may be observed for several orbitals, particularly in the majority channel. For the d_{z^2} and the $d_{x^2-y^2}$ orbitals, this may occur because they can hybridize with each other and as such are not eigenchannels, as also observed in the VASP calculations in Supplementary Note 6. For the d_{yz} orbital, this is most likely caused by the hybridization with the substrate which also displays peaks in the DOS in the same energy range (Supplementary Fig. 10b).

The connection between the features in the DOS and the parameters K^μ and $J^\mu S/2$ is given by the expressions

$$\tilde{J}^\mu = \frac{J^\mu S}{2}\pi\rho_0(E = 0) = -\pi\Gamma^\mu \frac{\mathcal{J}^\mu}{\left(\varepsilon_d^\mu\right)^2 - (\mathcal{J}^\mu)^2} 2S, \tag{5}$$

$$\widetilde{K}^{\mu} = K^{\mu}\pi\rho_0(E=0) = -\pi\Gamma^{\mu}\frac{\varepsilon_d^{\mu}}{\left(\varepsilon_d^{\mu}\right)^2 - (\mathcal{J}^{\mu})^2}, \tag{6}$$

where $\rho_0(E=0)$ is the DOS in the substrate at the Fermi level, $\pi\Gamma^{\mu}$ is the half-width at half-maximum of the resonance peaks characterizing the hybridization, and $\varepsilon_d^{\mu} \pm \mathcal{J}^{\mu}$ are the positions of the peaks in the minority and majority channels, respectively, the splitting of which leads to the emergence of the magnetic moment. Note that analogous expressions may be derived based on the Schrieffer–Wolff transformation[10,16,19],

$$\tilde{J}^{\mu} = \pi\Gamma^{\mu}\frac{U}{\tilde{\varepsilon}_d^{\mu}\left(\tilde{\varepsilon}_d^{\mu} + U\right)}2S, \tag{7}$$

$$\widetilde{K}^{\mu} = -\pi\Gamma^{\mu}\frac{2\tilde{\varepsilon}_d^{\mu} + U}{\tilde{\varepsilon}_d^{\mu}\left(\tilde{\varepsilon}_d^{\mu} + U\right)}, \tag{8}$$

where $\tilde{\varepsilon}_d^{\mu}$ and $\tilde{\varepsilon}_d^{\mu} + U$ are the energies of the first and the second electrons on the localized *d* level, the latter being increased due to the Coulomb repulsion. Supplementary Eq. (5) and (6) were deemed more suitable for describing the *ab initio* results, since the exchange–correlation magnetic field in the local spin density approximation of density functional theory breaks the spin symmetry, unlike the interacting Anderson impurity model on which Supplementary Eq. (7) and (8) are based. It is important to observe that Supplementary Eq. (5) and (6) hold under the assumptions that (i) the scattering channels may be treated together as a single spin of size S, (ii) the density of states in the substrate is a constant in the energy range of the order of ε_d^{μ} and $\mathcal{J}^{\mu}$, and (iii) the shape of the scattering potential is point-like (i.e., $\psi_{\mu}(\mathbf{k}) \equiv 1$). Under these assumptions, the energies of the Shiba states may be calculated as[20,21]

$$E_{\mathrm{YSR}}^{\mu} = \pm\Delta\frac{(\tilde{J}^{\mu})^2 - \left(\widetilde{K}^{\mu}\right)^2 - 1}{\sqrt{\left[(\tilde{J}^{\mu})^2 - \left(\widetilde{K}^{\mu}\right)^2 - 1\right]^2 + (2\tilde{J}^{\mu})^2}}. \tag{9}$$

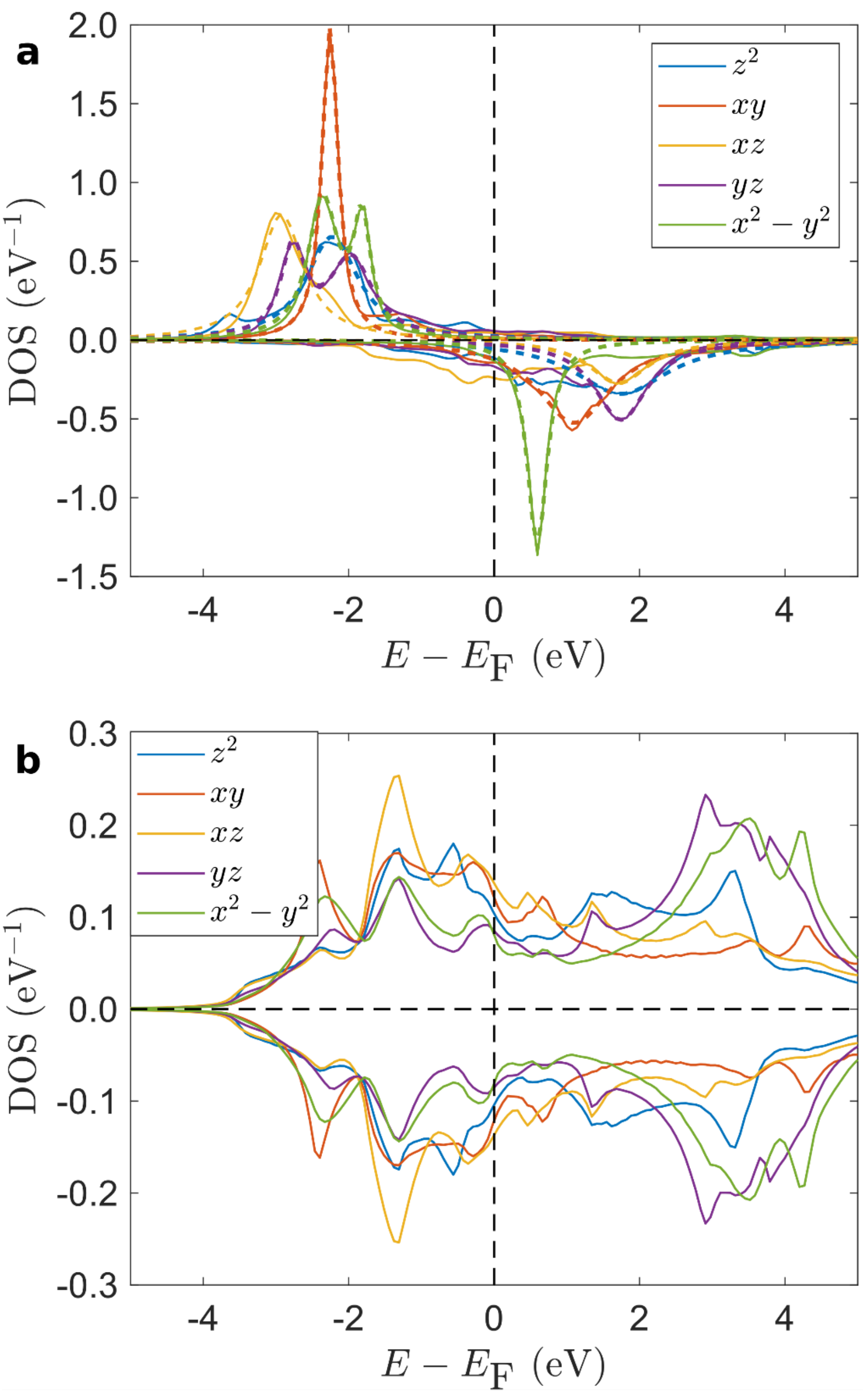

Supplementary Figure 10 | Density of states in the Mn adatom and in the Nb(110) surface layer. The density of states was calculated from the SKKR method with an imaginary part of the energy of 68 meV. The projection of the DOS on the real *d* orbitals is plotted for **a**, the Mn adatom and for **b**, the top Nb layer (solid curves). The majority and minority spin channels are shown with positive and negative values in the DOS, respectively. Dashed curves in panel **a** denote the Lorentzian curves fitted in the vicinity of the peak positions, used to extract the parameters in Supplementary Eq. (5) and (6).

The results obtained from Supplementary Eq. (9) are displayed in Supplementary Fig. 11, where the spin size S was used as a scaling parameter. The parameters ε_d^μ and $\mathcal{J}^\mu$ were extracted by the peak positions of Lorentzian peaks fitted on the DOS curves shown by dashed lines in Supplementary Fig. 10a. The value of $\pi\Gamma^\mu$ was calculated by determining the width of the Lorentzians, subtracting the artificial broadening of 68 meV introduced in the *ab initio* calculations, and linearly interpolating its value to the Fermi level between the positive and negative energy peaks. From the two peaks of the $d_{x^2-y^2}$ and d_{yz} orbitals in the majority spin channel, the one closer to the Fermi level was selected for the former and the one further away for the latter. It can be deduced from Supplementary Fig. 11, assuming a sufficiently strong coupling such that all Shiba states have crossed the Fermi level, that their energetical order based on the calculations agrees with the experimental one. Note that the $d_{x^2-y^2}$ state is neglected since only four states were observed experimentally. A quantitative agreement of the Shiba state energies with the experimental values requires assuming $2S = 8 - 10$ for the four selected orbitals. Considering that the assumption of a constant density of states in the substrate in a wide energy range is not supported by the calculations (see Supplementary Fig. 10b) and that the model described by Supplementary Eq. (9) does not take into account the shapes of the scattering potential or of the Fermi surface, we decided to use this description only for a qualitative evaluation of the Shiba states; see refs.[10,22] for similar arguments. Based on these calculations, we concluded that all of the Shiba states are in the limit of strong coupling, which yields the correct order of the Shiba states compared to the experimental situation, and the numerical values of K^μ and $J^\mu S/2$ were selected to reproduce the Shiba state positions assuming they already crossed the Fermi level.

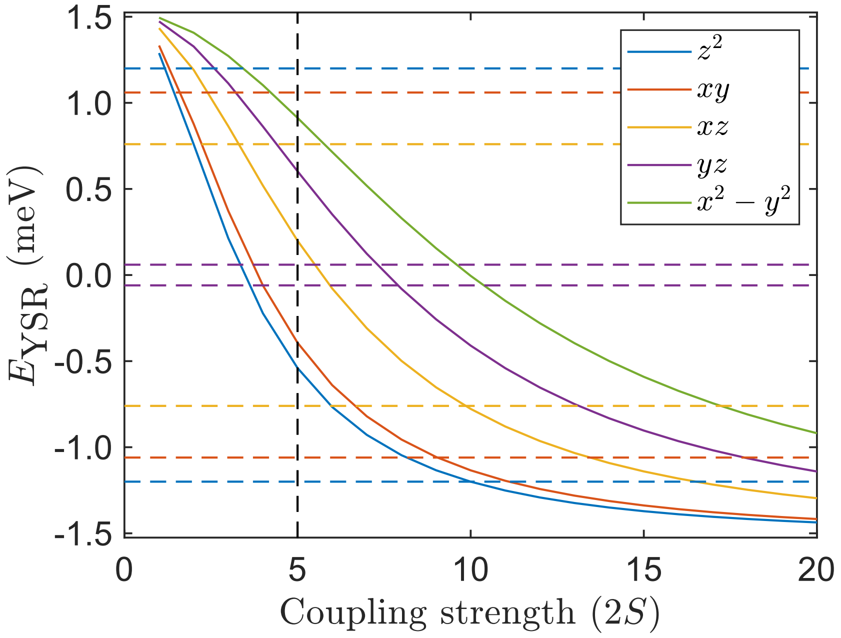

Supplementary Figure 11 | Energy of Shiba states determined from the SKKR calculations. Continuous lines denote results from Supplementary Eq. (9), with the parameters extracted as described in the text. Horizontal dashed lines denote the experimentally determined positions of the Shiba states, both at positive and negative energy. Shiba states coming from the same orbitals are denoted by the same color in the theoretical curves and in the experimental lines. The corresponding pairs of the Shiba states for the theoretical model are not plotted for simplicity. The total spin of the adatom S was used as a scaling parameter, with the expected value $2S = 5$ for Mn based on Hund's first rule denoted by a black dashed vertical line.

Supplementary Note 9. Hybridization of Shiba states in the dimers

Here we discuss the dependence of the splitting of the Shiba states on the angle between the spins and on the strength of the spin–orbit coupling. We consider the $\sqrt{3}a/2 - [1\bar{1}1]$ dimer, and determine the energies of the Shiba states from the condition

$$\det\left[I_4 - \sum_{\mathbf{k}''} \Psi_\mu^*(\mathbf{k}'') G_0(z, \mathbf{k}'') \Psi_\mu(\mathbf{k}'') \frac{1}{N} \left(K^\mu \tau^z - \frac{J^\mu S}{2} S_\mu^\alpha \sigma_4^\alpha \tau^z\right)\right] = 0, \quad (10)$$

cf. Eq. (9) in the Methods section of the main text. Only a single orbital is considered on each adatom in every calculation, thereby neglecting the hybridizations which would occur between different orbitals due to the reduced symmetry. As shown in Supplementary Fig. 12, in the absence of spin–orbit coupling the splitting between the energy

positions of the Shiba states monotonically decreases with the angle between the adatom spins, and completely disappears for an antiferromagnetic alignment as expected based on the arguments given in the main text. The only exception to the monotonic decrease can be observed for the δ state, where one of the hybridized orbitals crosses the Fermi level if the angle between the spins is below 140°. Note that the splitting of the β states is significantly smaller than for all the other cases in this specific dimer.

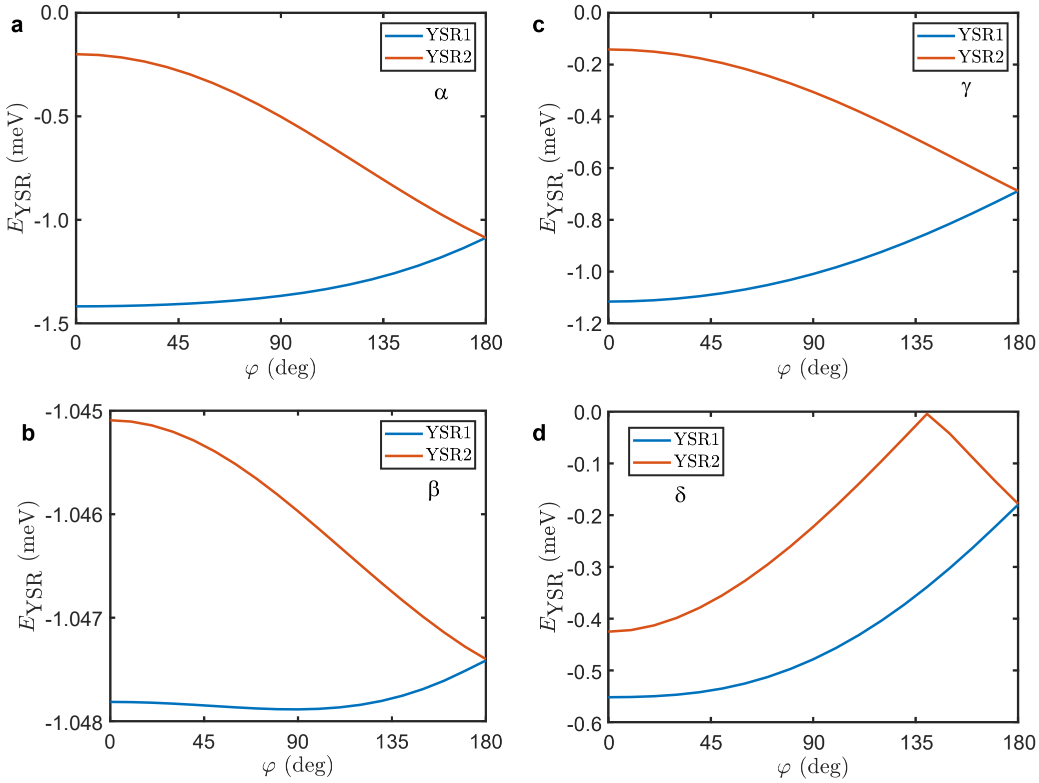

Supplementary Figure 12 | Hybridization of Shiba states in the $\sqrt{3}\mathrm{a}/2 - [1\bar{1}1]$ dimer without spin–orbit coupling. The scattering parameters are taken from Supplementary Table 2. The orientation of the spins is $\mathbf{S}_1 = (0,0,1)$ and $\mathbf{S}_2 = (\sin\varphi, 0, \cos\varphi)$. The Shiba state positions were calculated separately for **a**, the α, **b**, the β, **c**, the γ and **d**, the δ states.

When spin–orbit coupling is taken into account, the degeneracy of the states in the AFM case is lifted, as shown in Supplementary Fig. 13. Remarkably, the splitting of the β states is considerably larger in the antiferromagnetic alignment than in the ferromagnetic one, and it almost vanishes in the latter case, similar to the result without spin–orbit coupling in Supplementary Fig. 12b. Conversely, the splitting in the ferromagnetic alignment is higher than in the antiferromagnetic one for the other three Shiba orbitals, but even here the angle for which the splitting is maximal differs between the states. This points towards the observation that the effect of spin–orbit coupling is not equivalent

to rotating the spin directions on the different sites, as is the case in the one-dimensional Rashba model; see, e.g., ref.[23].

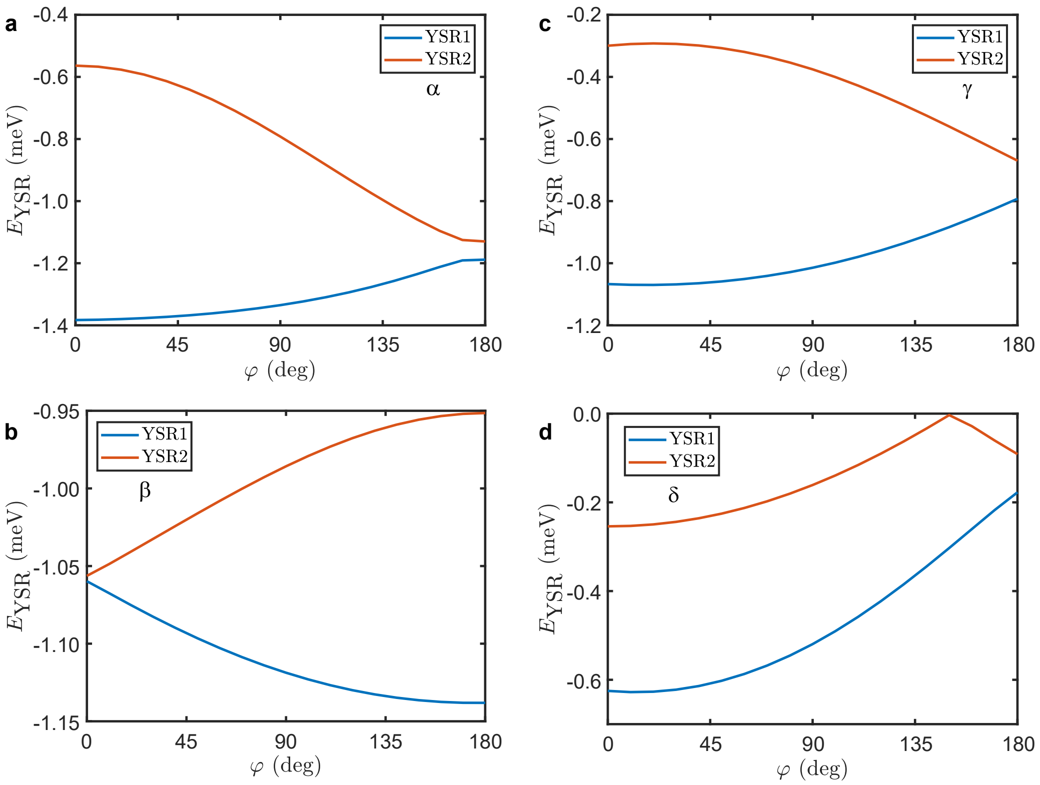

Supplementary Figure 13 | Hybridization of Shiba states in the $\sqrt{3}\mathbf{a}/2-[1\bar{1}1]$ dimer with spin–orbit coupling. The scattering parameters are taken from Supplementary Table 1. The orientation of the spins is $\mathbf{S}_1=(0,0,1)$ and $\mathbf{S}_2=(\sin\varphi, 0, \cos\varphi)$. The Shiba state positions were calculated separately for **a**, the α, **b**, the β, **c**, the γ and **d**, the δ states.

dimer	φ_0 (°)
$\sqrt{3}a/2-[1\bar{1}1]$	179.462
$\sqrt{2}a-[1\bar{1}0]$	179.931
$2a-[001]$	172.244

Supplementary Table 3 | Ground state angles between the spins in the different dimers from SKKR calculations.

In order to separate the effects of the non-collinear spin alignment and the Rashba term in the Hamiltonian on the splitting of the Shiba states, both of which are caused by the spin–orbit coupling in a non-centrosymmetric structure, the SKKR calculations discussed in Supplementary Note 8 were extended to dimers. The parameters of a classical spin model were extracted based on the spin-cluster expansion[24,25], and the ground state was found based on this spin model by energy minimization. The ground state angle between the spins is summarized in Supplementary Table 3 for the three dimers discussed in the paper. The calculations predict an almost AFM alignment for all three dimers, which agrees with the experimental results for the $\sqrt{3}\mathrm{a}/2-[1\bar{1}1]$ and the $2\ \mathrm{a}-[001]$ dimers, but disagrees for the $\sqrt{2}\ \mathrm{a}-[1\bar{1}0]$ dimer. The deviation from the collinear alignment is below 10° in all cases and only around 0.5° for the $\sqrt{3}\mathrm{a}/2-[1\bar{1}1]$ dimer, meaning that the Dzyaloshinsky–Moriya interaction causing the non-collinearity is weak in the system. Comparing these small angles to the calculation results in Supplementary Fig. 12, it is clear that the non-collinear alignment itself is insufficient for explaining the experimentally observed magnitude of the splittings of the Shiba states.

It is shown in Supplementary Fig. 14 how the splitting of the Shiba states in the antiferromagnetic alignment depends on the strength of the Rashba parameter t_{R}. In the absence of spin–orbit coupling, the states in the dimer are degenerate, although their energy position is slightly shifted compared to Supplementary Fig. 12 due to the different choice of the scattering parameters. As described in Supplementary Note 6, introducing these different scattering parameters was necessary because the presence of spin–orbit coupling changes the DOS at the Fermi level, which influences the Shiba state positions; see, e.g., Supplementary Eq. (5), (6) and (9) for a simple example. This modulation of the DOS is enhanced by the limited resolution in reciprocal space and the energy cut-off restricting the states close to the Fermi level, which may partially explain the considerable oscillations of the Shiba state energies in Supplementary Fig. 14 as the spin–orbit coupling is changed. The numerical accuracy is expected to be improved by considering a finer $\mathbf{k}$ mesh or a larger energy cut-off. The available data already supports that the effect of the single Rashba coupling parameter is not uniform on the different orbitals, as already discussed above for Supplementary Fig. 13. For example, the γ states are almost degenerate for t_{R}=10 meV, while the splitting is almost maximal at this value for the other orbitals.

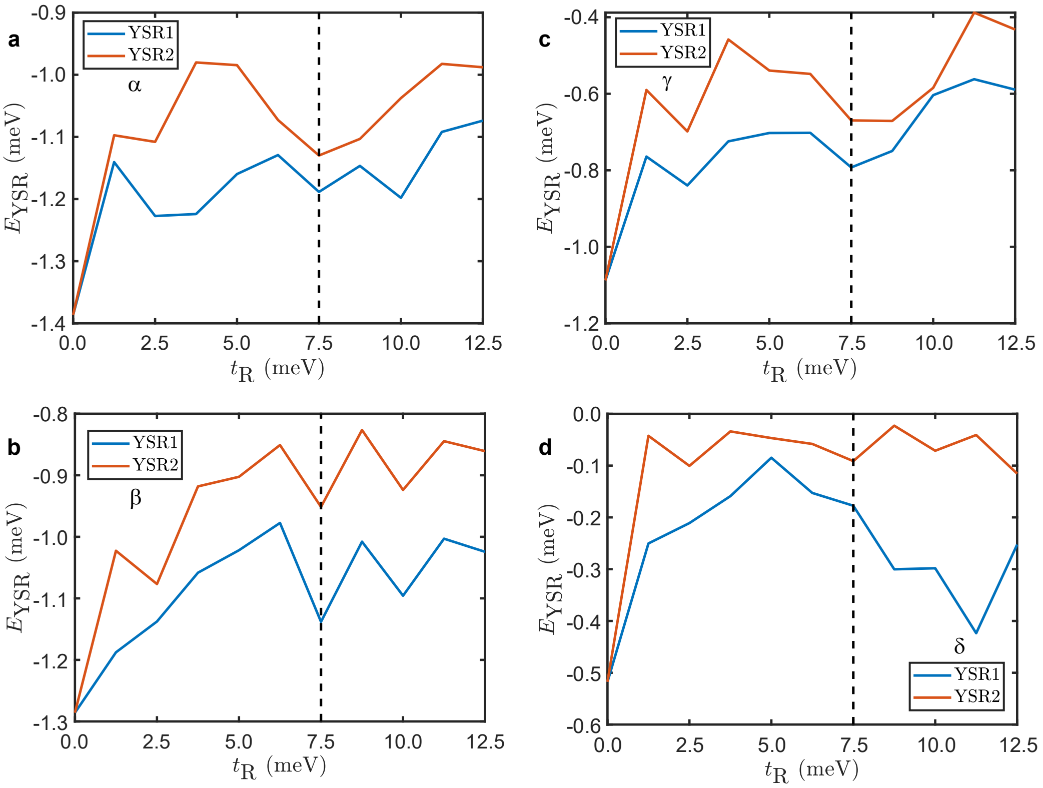

Supplementary Figure 14 | Hybridization of Shiba states in the $\sqrt{3}\mathbf{a}/2-[1\bar{1}1]$ dimer as a function of spin–orbit coupling. The scattering parameters are taken from Supplementary Table 1. An out-of-plane antiferromagnetic orientation of the spins was assumed. The Shiba state positions were calculated separately for **a**, the α, **b**, the β, **c**, the γ and **d**, the δ states. Dashed vertical lines denote the value of the Rashba coupling used in Figs. 4f-n in the main text.

Supplementary Note 10. Symmetry analysis of Shiba state hybridization

The possible hybridizations between Shiba states with different orbitals may be deduced based on group theoretical arguments. The character table of the $C_{2\mathrm{v}}$ point group is given in Supplementary Table 4. In this specific case, the character expresses whether the selected *d* orbital is invariant under the selected symmetry operation (1) or it changes sign under it (-1). The possible symmetry operations include the identity transformation E, a 180° rotation C_2 around the *z* axis, and mirroring σ_{v} on the *xz* and *yz* planes, respectively. The transformations are illustrated in Supplementary Figs. 15a-d for the d_{xy} orbital, which changes sign under both mirror operations but is invariant under the two-fold rotation. There are four possible different ways the orbitals may transform under the action of the group, known as irreducible representations (irreps). Since the Hamiltonian possesses a $C_{2\mathrm{v}}$ symmetry, all of its eigenstates, including the Shiba states, belong to one of the irreps, similarly to odd and even eigenfunctions in one dimension. Orbitals

belonging to different irreps are always orthogonal and cannot hybridize, but atomic orbitals belonging to the same irrep may hybridize, meaning that the eigenstates of the Hamiltonian will become a linear combination of these atomic orbitals. This can be observed for the d_{z^2} and $d_{x^2-y^2}$ orbitals in the present system, both of which belong to the A_1 irrep.

irrep	E	$C_2\ (z)$	$\sigma_v\ (xz)$	$\sigma_v\ (yz)$	*d* orbitals
A_1	1	1	1	1	x^2-y^2, z^2
A_2	1	1	-1	-1	*xy*
B_1	1	-1	1	-1	*xz*
B_2	1	-1	-1	1	*yz*

Supplementary Table 4 | Character table of the C_{2v} point group.

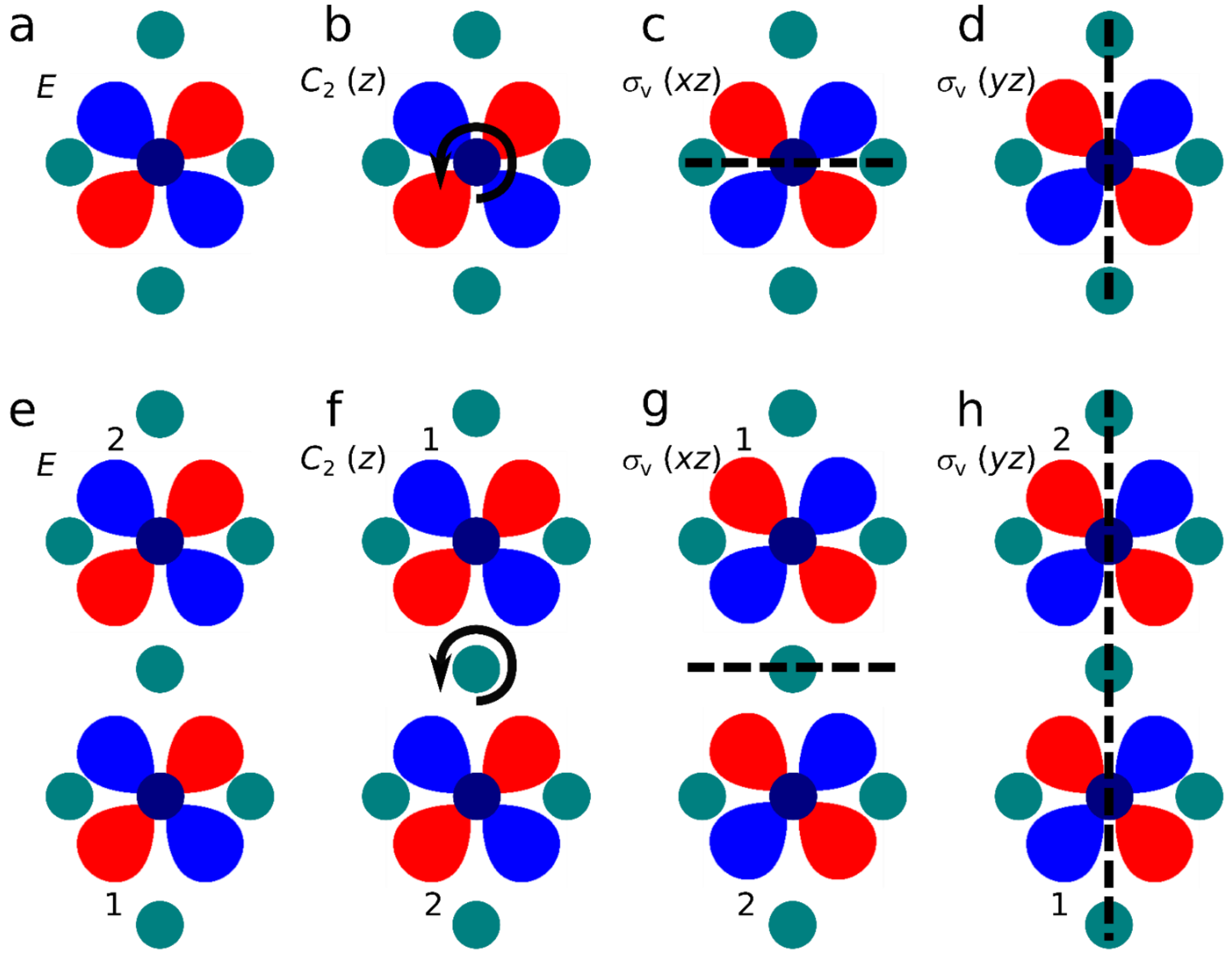

Supplementary Figure 15 | Transformation of the orbitals under point group operations. a-d Transformation of an orbital with d_{xy} shape under the symmetry operations included in Supplementary Table 4. Green circles denote atomic positions of Nb atoms. Red and blue lobes indicate positive and negative signs of the wave functions of a Mn adatom, respectively. **e-h** Transformation of two orbitals with d_{xy} shape in a $\sqrt{2}\mathrm{a}-[1\bar{1}0]$ dimer arrangement. The numbers 1 and 2 denote the different atomic positions.

The shapes of the Shiba states in the dimers may be deduced by describing them as linear combinations of the adatom orbitals. Under the symmetry transformations given in Supplementary Table 4, both orbitals may stay invariant (1), reverse sign (-1), or move to a different lattice site (0). This is illustrated in Supplementary Figs. 15e-h by considering a d_{xy} orbital on both adatoms in the $\sqrt{2}\mathrm{a}-[1\bar{1}0]$ dimer: the wave functions are invariant under E ($\chi=2$), they change sign under mirroring σ_{v} ($\chi=-2$) on the *yz* plane, and their positions are exchanged for the twofold rotation and mirroring on the *xz* plane ($\chi=0$). Summing up these numbers over the two orbitals give the characters of the selected symmetry transformation, which are listed in Supplementary Table 5. All of these characters, considered as vectors over the different symmetry operations, may be written as linear combinations of the irreps, as given in the last column of Supplementary Table 5. It can be seen that all pairs of orbitals reduce to a pair of bonding (A_1, B_1) and antibonding (A_2, B_2) states, which are even and odd under mirroring on the *xz* plane between the two adatoms, respectively. Eigenstates belonging to the same irreps may still hybridize with each other, meaning that the d_{z^2}, $d_{x^2-y^2}$ and d_{yz} orbitals localized at different adatoms will combine, but they will remain orthogonal to the states stemming from the d_{xy} and d_{xz} orbitals.

adatom orbital	E	$C_2\ (z)$	$\sigma_{\mathrm{v}}\ (xz)$	$\sigma_{\mathrm{v}}\ (yz)$	irreps
*x*²-*y*², *z*²	2	0	0	2	A_1+B_2
xy	2	0	0	-2	A_2+B_1
xz	2	0	0	-2	A_2+B_1
yz	2	0	0	2	A_1+B_2

Supplementary Table 5 | Reduction of atomic orbitals into irreducible representations for the $\sqrt{2}\mathbf{a}-[\mathbf{1\bar{1}0}]$ dimer. The same type of orbital is assumed on both adatoms in the dimer.

In the $\sqrt{3}\mathrm{a}/2-[1\bar{1}1]$ dimer, the adatom positions reduce the point group symmetry to C_2, with the character table given in Supplementary Table 6. The characters of pairs of atomic orbitals localized at different positions in the dimer have to be calculated according to this symmetry group, and then reduced to irreps, as shown in Supplementary Table 7. In this case, again all pairs of orbitals split into bonding (A) and antibonding (B) states, the latter having a node at the position where the $C_2\ (z)$ axis crosses the surface. Since all orbitals reduce to the same pair of irreps, any two of them may hybridize for this type of dimer.

irrep	E	$C_2\ (z)$	*d* orbitals
A	1	1	*xy*, *x*²-*y*², *z*²
B	1	-1	*xz*, *yz*

Supplementary Table 6 | Character table of the C_2 point group.

adatom orbital	E	$C_2\ (z)$	irreps
x^2-y^2, z^2	2	0	A+B
xy	2	0	A+B
xz	2	0	A+B
yz	2	0	A+B

Supplementary Table 7 | Reduction of atomic orbitals into irreducible representations for the $\sqrt{3}\mathbf{a}/2-[1\bar{1}\bar{1}]$ dimer. The same type of orbital is assumed on both adatoms in the dimer.

The above symmetry considerations did not take into account the spin degree of freedom, and they may only be applied if exchanging the atomic positions does not modify the spin configuration (i.e., for ferromagnetic dimers), and in the absence of spin–orbit coupling. Spin–orbit coupling mixes together the different atomic orbitals: for example, a d_{xy} orbital (with magnetic quantum number $m_l = \pm 2$) with spin pointing down ($m_l = -1/2$) is linearly combined with the d_{xz} orbital ($m_l = \pm 1$) with spin pointing up ($m_l = 1/2$) to form an eigenstate of the total angular momentum. Since the orbital part of the up and down spin components transforms differently under the symmetry operations (in the above example, consider the $C_2\ (z)$ rotation in Supplementary Table 6), antibonding states where the total density of states including contributions from both spin channels completely disappears at certain points or along specific lines both for positive and negative energy Shiba states may no longer be strictly defined. This is not immediately apparent for the ferromagnetic dimers if spin–orbit coupling is weak, but it strongly influences the states in antiferromagnetic dimers.

Supplementary Note 11. Complementary calculated Shiba states

Supplementary Figs. 16 to 18 show the calculated positive-bias counterparts of the two-dimensional maps of the LDOS of a Mn adatom (Supplementary Fig. 16), of a $\sqrt{2}\mathrm{a}-[1\bar{1}0]$ Mn dimer (Supplementary Fig. 17), and of a $\sqrt{3}\mathrm{a}/2-[1\bar{1}1]$ Mn dimer (Supplementary Fig. 18). They can be compared to their negative-bias counterparts given in the main text (Figs. 1k-n, Figs. 2l-s, and Figs. 4b-e and g-n, respectively). For the single adatom and the FM-coupled $\sqrt{2}\mathrm{a}-[1\bar{1}0]$ Mn dimer the spatial distributions of the LDOS at negative and positive bias voltages have very similar shapes. In contrast, for the AFM-coupled $\sqrt{3}\mathrm{a}/2-[1\bar{1}1]$ Mn dimer, the shapes are less similar and each Shiba state at positive energy appears rather symmetric compared to their negative-energy partners which are rather antisymmetric.

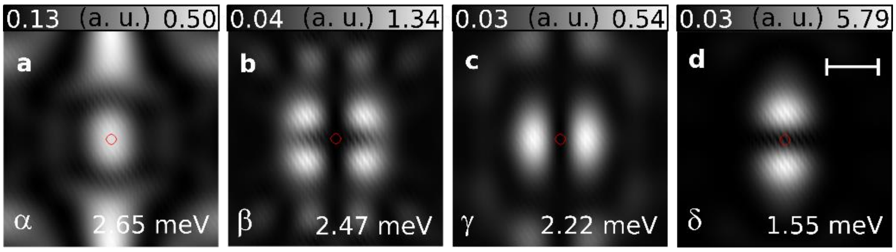

Supplementary Figure 16 | Calculated positive-bias LDOS of a Mn adatom on Nb(110). **a-d**, Positive-bias counterparts of the calculated two-dimensional maps of the LDOS in Figs. 1k-n in the main text of a single Mn adatom taken at the bias voltages as indicated. Red circles denote the position of the adatom. The white scale bar has a length of 500 pm.

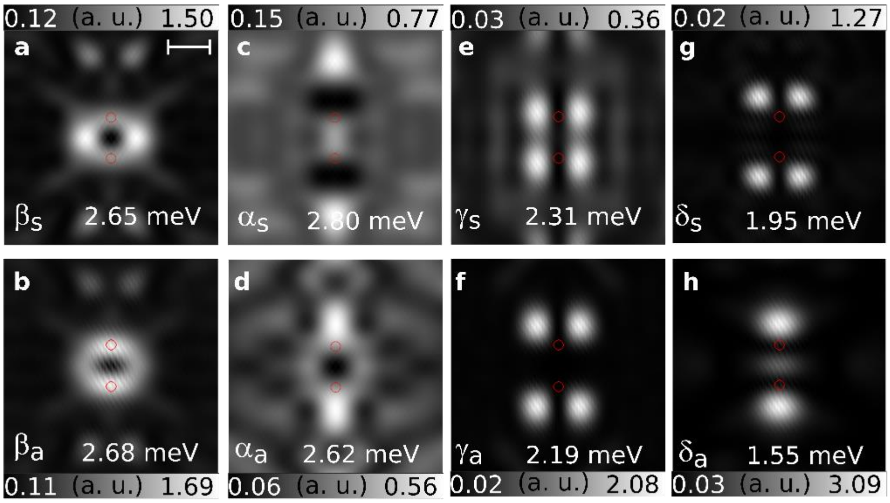

Supplementary Figure 17 | Calculated positive-bias LDOS of a FM-coupled $\sqrt{2}\mathbf{a}-[1\bar{1}0]$ Mn dimer. a-h, Positive-bias counterparts of the calculated two-dimensional maps of the LDOS in Figs. 2l-s in the main text taken at the bias voltages as indicated. Red circles denote the locations of the Mn adatoms in the dimer. The white scale bar has a length of 500 pm.

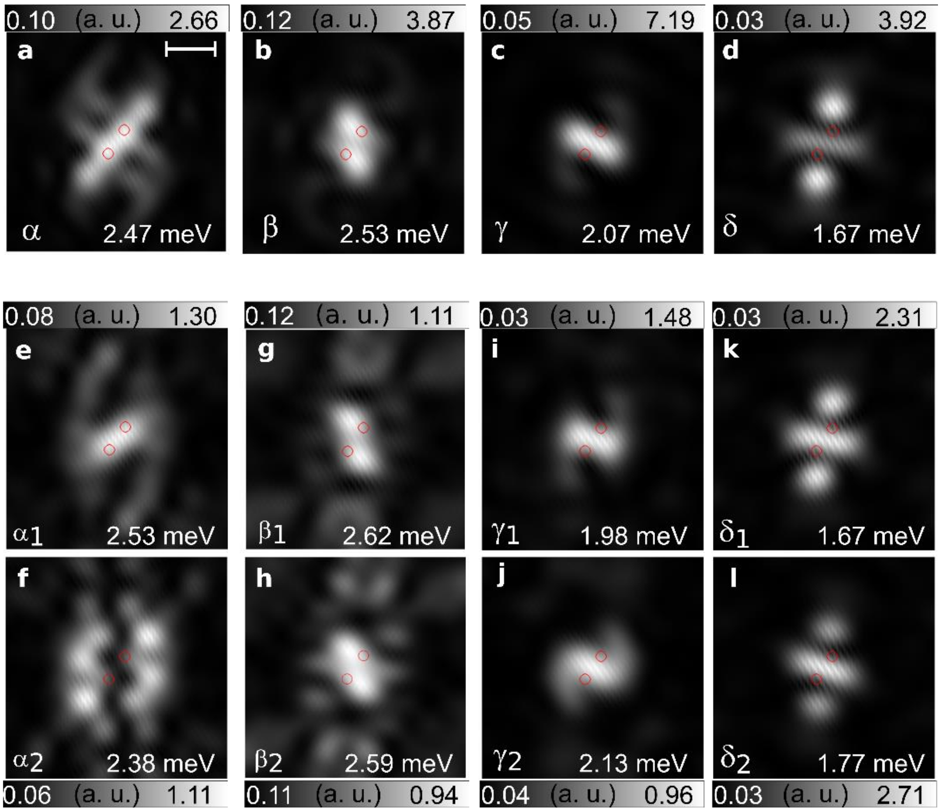

Supplementary Figure 18 | Calculated positive-bias LDOS of an AFM-coupled $\sqrt{3}\mathbf{a}/2-[1\bar{1}1]$ Mn dimer. Positive-bias counterparts of the two-dimensional maps of the LDOS in Figs. 4b-e and g-n in the main text at the indicated bias voltages **a**-**d**, without and **e**-**l**, with SOC. Red circles denote the positions of the adatoms in the dimer. The white scale bar has a length of 500 pm.

References

1. Odobesko, A. B. *et al.* Preparation and electronic properties of clean superconducting Nb(110) surfaces. *Phys. Rev. B* **99**, 115437 (2019).

2. Stroscio, J. A. & Celotta, R. J. Controlling the dynamics of a single atom in lateral atom manipulation. *Science* **306**, 242–247 (2004).

3. Pillet, J.-D. *et al.* Andreev bound states in supercurrent-carrying carbon nanotubes revealed. *Nat. Phys.* **6**, 965–969 (2010).

4. Schneider, L., Beck, P., Wiebe, J. & Wiesendanger, R. Atomic-scale spin-polarization maps using functionalized superconducting probes. *Sci. Adv.* **7**, eabd7302 (2021).

5. Wiesendanger, R. Spin mapping at the nanoscale and atomic scale. *Rev. Mod. Phys.* **81**, 1495–1550 (2009).

6. Kresse, G. & Furthmüller, J. Efficiency of ab-initio total energy calculations for metals and semiconductors using a plane-wave basis set. *Comput. Mater. Sci.* **6**, 15–50 (1996).

7. Kresse, G. & Furthmüller, J. Efficient iterative schemes for ab initio total-energy calculations using a plane-wave basis set. *Phys. Rev. B* **54**, 11169–11186 (1996).

8. Hafner, J. Ab-initio simulations of materials using VASP: density-functional theory and beyond. *Journal of Computational Chemistry* **29**, 2044–2078 (2008).

9. Perdew, J. P., Burke, K. & Ernzerhof, M. Generalized gradient approximation made simple. *Phys. Rev. Lett.* **77**, 3865–3868 (1996).

10. Choi, D.-J. *et al.* Mapping the orbital structure of impurity bound states in a superconductor. *Nat. Commun.* **8**, 15175 (2017).

11. WaveTrans. Available at: https://www.andrew.cmu.edu/user/feenstra/wavetrans/. (Accessed: 30th September 2020).

12. Feenstra, R. M. *et al.* Low-energy electron reflectivity from graphene. *Phys. Rev. B* **87**, 041406 (2013).

13. Szunyogh, L., Újfalussy, B. & Weinberger, P. Magnetic anisotropy of iron multilayers on Au(001): first-principles calculations in terms of the fully relativistic spin-polarized screened KKR method. *Phys. Rev. B* **51**, 9552–9559 (1995).

14. Faulkner, J. S. & Stocks, G. M. Calculating properties with the coherent-potential approximation. *Phys. Rev. B* **21**, 3222–3244 (1980).

15. Gonis, A. Green functions for ordered and disordered systems. (North-Holland, 1992).

16. Moca, C. P., Demler, E., Jankó, B. & Zaránd, G. Spin-resolved spectra of Shiba multiplets from Mn impurities in MgB_2. *Phys. Rev. B* **77**, 174516 (2008).

17. Lazarovits, B., Szunyogh, L. & Weinberger, P. Fully relativistic calculation of magnetic properties of Fe, Co, and Ni adclusters on Ag(100). *Phys. Rev. B* **65**, 104441 (2002).

18. Lloyd, P. Wave propagation through an assembly of spheres: III. The density of states in a liquid. *Proc. Phys. Soc.* **90**, 217–231 (1967).

19. Schrieffer, J. R. & Wolff, P. A. Relation between the Anderson and Kondo Hamiltonians. *Phys. Rev.* **149**, 491–492 (1966).

20. Shiba, H. Classical spins in superconductors. *Prog. Theor. Phys.* **40**, 435–451 (1968).

21. Rusinov, A. I. Theory of gapless superconductivity in alloys containing paramagnetic impurities. *Sov. Phys. JETP* **29**, 1101 (1969).

22. Korytár, R. & Lorente, N. Multi-orbital non-crossing approximation from maximally localized Wannier functions: the Kondo signature of copper phthalocyanine on Ag(100). *J. Phys. Condens. Matter* **23**, 355009 (2011).

23. Braunecker, B., Japaridze, G. I., Klinovaja, J. & Loss, D. Spin-selective Peierls transition in interacting one-dimensional conductors with spin-orbit interaction. *Phys. Rev. B* **82**, 045127 (2010).

24. Drautz, R. & Fähnle, M. Spin-cluster expansion: parametrization of the general adiabatic magnetic energy surface with ab initio accuracy. *Phys. Rev. B* **69**, 104404 (2004).

25. Lászlóffy, A., Udvardi, L. & Szunyogh, L. Atomistic simulation of finite-temperature magnetism of nanoparticles: application to cobalt clusters on Au(111). *Phys. Rev. B* **95**, 184406 (2017).
